# Supplementary material for: miR-125-chinmo pathway regulates dietary restriction-dependent enhancement of lifespan in Drosophila
Source: eLife. 2021 Jun 8;10:e62621. doi: 10.7554/eLife.62621 (PMC8233039; doi:10.7554/eLife.62621)
Supplement: Figure 1—source data 1. [file elife-62621-fig1-data1.docx]

**Figure 1-source data 1.** Lifespan analysis of *let-7-Complex^hyp^, let-7-Complex^hyp^* rescue, *let-7-Complex^null^* *rescue*, *ΔmiR-100*, *Δlet-7* and *ΔmiR-125* mutant lines.

|  | **Lifespan (Days)** | | **p value** | **χ^2^** |
| --- | --- | --- | --- | --- |
| #Experiment 1 | Maximum  (Number of flies) | Median |  |  |
| *w^1118^; let-7-C^GKI^/ let-7-C^KO2^, P{neoFRT}40A; P {w+, let-7-Cp^3.3kb^::cDNA}VK00033 / {v+, let-7-C }attP2 AL* | 60 (93) | 36 | 0.00E+00 | 92.72 |
| *w^1118^; let-7-C^GKI^/ let-7-C^KO2^, P{neoFRT}40A; P {w+, let-7-Cp^3.3kb^::cDNA}VK00033 / {v+, let-7-C }attP2 DR* | 82 (97) | 62 |  |  |
| *w^1118^; let-7-C^GKI^/ let-7-C^KO2^, P{neoFRT}40A; P {w+, let-7-Cp^3.3kb^::cDNA}VK00033 / {v+, let-7-C ^Δlet-7-C miRNAs^} attP2 AL* | 36 (111) | 24 | 1.30E-06 | 23.45 |
| *w^1118^; let-7-C^GKI^/ let-7-C^KO2^, P{neoFRT}40A; P {w+, let-7-Cp^3.3kb^::cDNA}VK00033 / {v+, let-7-C ^Δlet-7-C miRNAs^} attP2 DR* | 42 (72) | 29 |  |  |
| *w^1118^; let-7-C^GKI^ / let-7-C^KO2^, P{neoFRT}40A; {v+, let-7-C} attP2/ + AL* | 68(160) | 38 | 0.00E+00 | 116.3 |
| *w^1118^; let-7-C^GKI^ / let-7-C^KO2^, P{neoFRT}40A; {v+, let-7-C} attP2 /+ DR* | 96(154) | 56 |  |  |
| *w^1118^; let-7-C^GKI^ / let-7-C^KO2^, P{neoFRT}40A; {v+, let-7-C ^ΔmiR-100^} attP2/ + AL* | 56(199) | 32 | 0.00E+00 | 43.62 |
| *w^1118^; let-7-C^GKI^ / let-7-C^KO2^, P{neoFRT}40A; {v+, let-7-C ^ΔmiR-100^} attP2/ + DR* | 76(185) | 38 |  |  |
| *w^1118^; let-7-C^GKI^ / let-7-C^KO2^, P{neoFRT}40A; {v+, let-7-C ^Δlet-7^} attP2 / + AL* | 42(86) | 19 | 0.0355 | 4.42 |
| *w^1118^; let-7-C^GKI^ / let-7-C^KO2^, P{neoFRT}40A; {v+, let-7-C ^Δlet-7^} attP2 / +DR* | 40(85) | 24 |  |  |
| *w^1118^; let-7-C^GKI^ / let-7-C^KO2^, P{neoFRT}40A; {v+, let-7-C ^ΔmiR-125^} attP2 / + AL* | 48(141) | 30 | 0.0384 | 4.29 |
| *w^1118^; let-7-C^GKI^ / let-7-C^KO2^, P{neoFRT}40A; {v+, let-7-C ^ΔmiR-125^} attP2 / + DR* | 52(122) | 32 |  |  |
|  | | | | |
| Experiment 2 |  |  |  |  |
| *w^1118^; let-7-C^GKI^/ let-7-C^KO2^, P{neoFRT}40A; P {w+, let-7-Cp^3.3kb^::cDNA}VK00033 / {v+, let-7-C }attP2 AL* | 40(114) | 26 | 0.00E+00 | 62.82 |
| *w^1118^; let-7-C^GKI^/ let-7-C^KO2^, P{neoFRT}40A; P {w+, let-7-Cp^3.3kb^::cDNA}VK00033 / {v+, let-7-C }attP2 DR* | 58(107) | 36 |  |  |
| *w^1118^; let-7-C^GKI^/ let-7-C^KO2^, P{neoFRT}40A; P {w+, let-7-Cp^3.3kb^::cDNA}VK00033 / {v+, let-7-C ^Δlet-7-C miRNAs^}attP2 AL* | 36(84) | 22 | 3.80E-08 | 30.27 |
| *w^1118^; let-7-C^GKI^/ let-7-C^KO2^, P{neoFRT}40A; P {w+, let-7-Cp^3.3kb^::cDNA}VK00033 / {v+, let-7-C ^Δlet-7-C miRNAs^}attP2 DR* | 44(75) | 30 |  |  |
| *w^1118^; let-7-C^GKI^ / let-7-C^KO2^, P{neoFRT}40A; {v+, let-7-C} attP2 / +AL* | 72(186) | 36 | 0.00E+00 | 48.93 |
| *w^1118^; let-7-C^GKI^ / let-7-C^KO2^, P{neoFRT}40A; {v+, let-7-C} attP2 / + DR* | 96(147) | 44 |  |  |
| *w^1118^; let-7-C^GKI^ / let-7-C^KO2^, P{neoFRT}40A; {v+, let-7-C ^ΔmiR-100^} attP2/ + AL* | 56(211) | 28 | 0.00E+00 | 103.38 |
| *w^1118^; let-7-C^GKI^ / let-7-C^KO2^, P{neoFRT}40A; {v+, let-7-C ^ΔmiR-100^} attP2/ + DR* | 82(196) | 46 |  |  |
| *w^1118^; let-7-C^GKI^ / let-7-C^KO2^, P{neoFRT}40A; {v+, let-7-C ^Δlet-7^} attP2 / + AL* | 40(86) | 20 | 0.0399 | 4.22 |
| *w^1118^; let-7-C^GKI^ / let-7-C^KO2^, P{neoFRT}40A; {v+, let-7-C ^Δlet-7^} attP2 / +DR* | 40(113) | 24 |  |  |
| *w^1118^; let-7-C^GKI^ / let-7-C^KO2^, P{neoFRT}40A; {v+, let-7-C ^ΔmiR-125^} attP2 / + AL* | 48(146) | 28 | 0.281 | 1.16 |
| *w^1118^; let-7-C^GKI^ / let-7-C^KO2^, P{neoFRT}40A; {v+, let-7-C ^ΔmiR-125^} attP2 / + DR* | 52(139) | 28 |  |  |

#Experiment 1 is represented in Figure 1H-M; p value calculated by log rank test; χ^2^, Chi^2^ calculated by Log rank test.
