# Supplementary material for: miR-125-chinmo pathway regulates dietary restriction-dependent enhancement of lifespan in Drosophila
Source: eLife. 2021 Jun 8;10:e62621. doi: 10.7554/eLife.62621 (PMC8233039; doi:10.7554/eLife.62621)
Supplement: Figure 1—figure supplement 2—source data 1. [file elife-62621-fig1-figsupp2-data1.docx]

**Figure 1-source data 2.** Lifespan analysis to test the effect of genetic background on lifespan

in the fly strains used in Figure 1.

|  |  | **Lifespan (Days)** | | **p value** | **χ^2^** |
| --- | --- | --- | --- | --- | --- |
| Experiment  No. | Genotype | Maximum  (Number of flies) | Median |  |  |
| 1^#^ | *w^1118^ AL* | 50(99) | 28 | 0.00E+00 | 156 |
|  | *w^1118^ DR* | 90(98) | 58 |  |  |
| 2 | *w^1118^ AL* | 54(156) | 22 | 0.00E+00 | 116.61 |
|  | *w^1118^ DR* | 82(162) | 40 |  |  |
|  |  |  |  |  |  |
| 1* | *let-7-C^KO2^, P{neoFRT}40 /+; +/+ AL* | 38(110) | 22 | 0.00E+00 | 60.19 |
|  | *let-7-C^KO2^, P{neoFRT}40 /+; +/+ DR* | 62(113) | 34 |  |  |
|  |  |  |  |  |  |
| 1* | *let-7-C^GKI^ /+; +/+ AL* | 64(106) | 44 | 0.00E+00 | 115.8 |
|  | *let-7-C^GKI^ /+; +/+ DR* | 106(112) | 70 |  |  |
|  |  |  |  |  |  |
| 1* | *let-7-C^GKI^ /+; P {v+, let-7-C} attP2*/+ *AL* | 54(102) | 36 | 3.5E-07 | 25.96 |
|  | *let-7-C^GKI^ /+; P {v+, let-7-C} attP2*/+ *DR* | 72(100) | 48 |  |  |
|  |  |  |  |  |  |
| 1* | *let-7-C^GKI^ /+; P {v+, let-7-C ^ΔmiR-100^} attP2*/+ *AL* | 38(103) | 12 | 0.00E+00 | 128.8 |
|  | *let-7-C^GKI^ /+; P {v+, let-7-C ^ΔmiR-100^} attP2*/+ *DR* | 72(79) | 50 |  |  |
|  |  |  |  |  |  |
| 1* | *let-7-C^GKI^ /+; P {v+, let-7-C ^Δlet-7^} attP2* /+ *AL* | 44(83) | 20 | 0.00E+00 | 87.91 |
|  | *let-7-C^GKI^ /+; P {v+, let-7-C ^Δlet-7^} attP2* /+ *DR* | 76(96) | 43 |  |  |
| 2 | *let-7-C^GKI^ /+; P {v+, let-7-C ^Δlet-7^} attP2* /+ *AL* | 42(151) | 24 | 0.00E+00 | 136.8 |
|  | *let-7-C^GKI^ /+; P {v+, let-7-C ^Δlet-7^} attP2* /+ *DR* | 52(139) | 40 |  |  |
|  |  |  |  |  |  |
| 1* | *let-7-C^GKI^ /+; P {v+, let-7-C ^ΔmiR-125^} attP2* /+ *AL* | 42(91) | 32 | 0.00E+00 | 76.01 |
|  | *let-7-C^GKI^ /+; P {v+, let-7-C ^ΔmiR-125^} attP2* /+ *DR* | 70(100) | 46 |  |  |
|  |  |  |  |  |  |
| 1* | *let-7-C^GKI^ / P {w+, let-7-Cp^3.3kb^::cDNA}VK00033* /+ *AL* | 42(139) | 28 | 0.00E+00 | 86.85 |
|  | *let-7-C^GKI^ / P {w+, let-7-Cp^3.3kb^::cDNA}VK00033* /+ *DR* | 62(160) | 40 |  |  |
| 1* | *let-7-C^GKI^ /+; P {v+, let-7-C ^Δlet-7-C^} attP2* /+ *AL* | 40(96) | 30 | 0.00E+00 | 65.50 |
|  | *let-7-C^GKI^ /+; P {v+, let-7-C ^Δlet-7-C^} attP2* /+ *DR* | 60(117) | 36 |  |  |

#*Experiment 1 for all background strains is depicted in Figure 1-supplement 2A^#^ and 2E-K*; p value calculated by log

rank test; χ^2^, Chi^2^ calculated by Log rank test.
