# Supplementary material for: miR-125-chinmo pathway regulates dietary restriction-dependent enhancement of lifespan in Drosophila
Source: eLife. 2021 Jun 8;10:e62621. doi: 10.7554/eLife.62621 (PMC8233039; doi:10.7554/eLife.62621)
Supplement: Figure 2—source data 1. [file elife-62621-fig2-data1.docx]

**Figure 2-source data 1.** Lifespan analysis of *rescue, chinmo^RNAi^* and *ΔmiR-125, chinmo^RNAi^* strains.

|  | **Lifespan (Days)** | | **p value**** | **χ^2^** |
| --- | --- | --- | --- | --- |
| *Experiment 1 | Maximum  (No. of flies) | Median |  |  |
| *w^1118^; let-7-C^GKI^ / let-7-C^KO2^, P{neoFRT}40A; {v+, let-7-C} attP2 / P{w+, UAS-chinmo^RNAi 148^}VK00033 AL* | 38(80) | 24 | 0.00E+00 | 37.35 |
| *w^1118^; let-7-C^GKI^ / let-7-C^KO2^, P{neoFRT}40A; {v+, let-7-C} attP2 / P{w+, UAS-chinmo^RNAi 148^}VK00033 DR* | 62(84) | 32 |  |  |
| *w^1118^; let-7-C^GKI^ / let-7-C^KO2^, P{neoFRT}40A; {v+, let-7-C ^ΔmiR-125^} attP2 / P{w+, UAS-chinmo^RNAi 148^}VK00033 AL* | 36(86) | 24 | 0.00E+00 | 57.71 |
| *w^1118^; let-7-C^GKI^ / let-7-C^KO2^, P{neoFRT}40A; {v+, let-7-C ^ΔmiR-125^} attP2 / P{w+, UAS-chinmo^RNAi 148^}VK00033* *DR* | 50(101) | 32 |  |  |
|  |  |  |  |  |
| Experiment 2 | | | | |
| *w^1118^; let-7-C^GKI^ / let-7-C^KO2^, P{neoFRT}40A; {v+, let-7-C} attP2 / P{w+, UAS-chinmo^RNAi 148^}VK00033 AL* | 42(183) | 30 | 0.00E+00 | 44.04 |
| *w^1118^; let-7-C^GKI^ / let-7-C^KO2^, P{neoFRT}40A; {v+, let-7-C} attP2 / P{w+, UAS-chinmo^RNAi 148^}VK00033 DR* | 80(251) | 34 |  |  |
| *w^1118^; let-7-C^GKI^ / let-7-C^KO2^, P{neoFRT}40A; {v+, let-7-C ^ΔmiR-125^} attP2 / P{w+, UAS-chinmo^RNAi 148^}VK00033 AL* | 32(105) | 22 | 0.00E+00 | 62.02 |
| *w^1118^; let-7-C^GKI^ / let-7-C^KO2^, P{neoFRT}40A; {v+, let-7-C ^ΔmiR-125^} attP2 / P{w+, UAS-chinmo^RNAi 148^}VK00033* *DR* | 40(98) | 28 |  |  |

*Experiment 1 is represented in Figure 2C-D; *p value calculated by log rank test; * χ^2^, Chi^2^ calculated by

Log rank test.
