# Supplementary material for: miR-125-chinmo pathway regulates dietary restriction-dependent enhancement of lifespan in Drosophila
Source: eLife. 2021 Jun 8;10:e62621. doi: 10.7554/eLife.62621 (PMC8233039; doi:10.7554/eLife.62621)
Supplement: Figure 2—source data 2. [file elife-62621-fig2-data2.docx]

**Figure 2-source data 2.** Lifespan analysis of*rescue, chinmo^1^* and *ΔmiR-125, chinmo^1^*strains.

|  | **Lifespan (Days)** | | **p value*** | **c^2^** |
| --- | --- | --- | --- | --- |
| *Experiment 1 | Maximum (Number of flies) | Median |  |  |
| *w^1118^; let-7-C^GKI^ / chinmo^1^, let-7-C^KO2^, P{neoFRT}40A; {v+, let-7-C} attP2 / + AL* | 76(110) | 40 | 0.00E+00 | 29.63 |
| *w^1118^; let-7-C^GKI^ / chinmo^1^, let-7-C^KO2^, P{neoFRT}40A; {v+, let-7-C} attP2 / + DR* | 96(105) | 50 |  |  |
| *w^1118^; let-7-C^GKI^ / chinmo^1^, let-7-C^KO2^, P{neoFRT}40A; {v+, let-7-C ^ΔmiR-125^} attP2 / +AL* | 64(114) | 34 | 0.00E+00 | 102.5 |
| *w^1118^; let-7-C^GKI^ / chinmo^1^, let-7-C^KO2^, P{neoFRT}40A; {v+, let-7-C ^ΔmiR-125^} attP2 / +* *DR* | 98(98) | 58 |  |  |
| Experiment 2 | | | | |
| *w^1118^; let-7-C^GKI^ / chinmo^1^, let-7-C^KO2^, P{neoFRT}40A; {v+, let-7-C} attP2 / + AL* | 84(128) | 38 | 0.00E+00 | 22.35 |
| *w^1118^; let-7-C^GKI^ / chinmo^1^, let-7-C^KO2^, P{neoFRT}40A; {v+, let-7-C} attP2 / + DR* | 98(147) | 46 |  |  |
| *w^1118^; let-7-C^GKI^ / chinmo^1^, let-7-C^KO2^, P{neoFRT}40A; {v+, let-7-C ^ΔmiR-125^} attP2 / +* *AL* | 56(85) | 28 | 0.00E+00 | 72.91 |
| *w^1118^; let-7-C^GKI^ / chinmo^1^, let-7-C^KO2^, P{neoFRT}40A; {v+, let-7-C ^ΔmiR-125^} attP2 / +* *DR* | 86(97) | 54 |  |  |
| Experiment 3 | | | | |
| *w^1118^; let-7-C^GKI^ / chinmo^1^, let-7-C^KO2^, P{neoFRT}40A; {v+, let-7-C} attP2 / + AL* | 78(127) | 32 | 0.00E+00 | 17.65 |
| *w^1118^; let-7-C^GKI^ / chinmo^1^, let-7-C^KO2^, P{neoFRT}40A; {v+, let-7-C} attP2 / + DR* | 96(182) | 36 |  |  |
| *w^1118^; let-7-C^GKI^ / chinmo^1^, let-7-C^KO2^, P{neoFRT}40A; {v+, let-7-C ^ΔmiR-125^} attP2 / +* *AL* | 60(95) | 24 | 0.00E+00 | 38.70 |
| *w^1118^; let-7-C^GKI^ / chinmo^1^, let-7-C^KO2^, P{neoFRT}40A; {v+, let-7-C ^ΔmiR-125^} attP2 / +* *DR* | 94(106) | 37 |  |  |

*Experiment 1 is represented in Figure 2; p value calculated by log rank test; ** **χ^2^, Chi^2^ calculated by Log rank test.
