# Supplementary material for: miR-125-chinmo pathway regulates dietary restriction-dependent enhancement of lifespan in Drosophila
Source: eLife. 2021 Jun 8;10:e62621. doi: 10.7554/eLife.62621 (PMC8233039; doi:10.7554/eLife.62621)
Supplement: Figure 2—figure supplement 1—source data 1. [file elife-62621-fig2-figsupp1-data1.docx]

**Figure 2-figure supplement 1-source data 1A. Lifespan analysis of flies encoding a single copy of the *UAS chinmo^RNAi^*transgene and a strain that is heterozygous for *let-7-C* and c*hinmo*.**

| Experiment^#^ | **Lifespan (Days)** | | **p value** | **χ^2^** |
| --- | --- | --- | --- | --- |
| Genotype | Maximum  (Number of flies) | Median |  |  |
| *+/+; UAS chinmo^RNAi^/+ DR-RU* | 92(96) | 56 | 0.2277 | 1.46 |
| *+/+; UAS chinmo^RNAi^/+ DR+RU* | 90(103) | 64 |  |  |
| *+/+; UAS chinmo^RNAi^/+ AL-RU* | 44(108) | 32 | 0.9711 | 0 |
| *+/+; UAS chinmo^RNAi^/+ AL+RU* | 44(99) | 32 |  |  |
| *+/+; UAS chinmo^RNAi^/+ DR-RU* | 92(96) | 56 | 0.00E+00 | 133.7 |
| *+/+; UAS chinmo^RNAi^/+ AL-RU* | 44(108) | 32 |  |  |
| *+/+; UAS chinmo^RNAi^/+ DR +RU* | 90(103) | 64 | 0.00E+00 | 151 |
| *+/+; UAS chinmo^RNAi^/+ AL+RU* | 44(99) | 32 |  |  |
|  | | | | |
| *chinmo^1^, let-7-C^KO2^/+; +/+ AL* | 60(104) | 38 | 0.00E+00 | 48.52 |
| *chinmo^1^, let-7-C^KO2^/+; +/+ DR* | 86(90) | 58 |  |  |

^#^ This experiment tests the effect of diet and ligand in the RNAi genetic background and the effect of

diet in a heterozygous background graph is depicted in Figure 2-supplement 1A-B; p value calculated

by log rank test; ** **χ^2^, Chi^2^ calculated by Log rank test.

**Figure 2- figure supplement 1-source data 1B.** **Cox proportional analysis of flies that encode a single copy of the *UAS chinmo^RNAi^*transgene.**

|  | **Risk factor** | **p value** |
| --- | --- | --- |
| *+/+; UAS chinmo^RNAi^/+* | Diet | 0 |
|  | Ligand | 0.722871 |
