# Supplementary material for: miR-125-chinmo pathway regulates dietary restriction-dependent enhancement of lifespan in Drosophila
Source: eLife. 2021 Jun 8;10:e62621. doi: 10.7554/eLife.62621 (PMC8233039; doi:10.7554/eLife.62621)
Supplement: Figure 3—source data 1. [file elife-62621-fig3-data1.docx]

**Figure 3-source data 1A.** Lifespan analysis of *FBGS> UAS chinmo^RNAi^* strain.

|  | **Lifespan (Days)** | | **p value** | **χ^2^** |
| --- | --- | --- | --- | --- |
| ^#^Experiment 1 | Maximum  (Number of flies) | Median |  |  |
| *FBGS/+; UAS chinmo^RNAi^/+ DR-RU* | 92(138) | 62 | 0.0408 | 4.185 |
| *FBGS/+; UAS chinmo^RNAi^/+ DR+RU* | 102(88) | 60 |  |  |
| *FBGS/+; UAS chinmo^RNAi^/+ AL-RU* | 72(97) | 42 | 5.40E-06 | 20.70 |
| *FBGS/+; UAS chinmo^RNAi^/+ AL+RU* | 80(98) | 50 |  |  |
| *FBGS/+; UAS chinmo^RNAi^/+ DR-RU* | 92(138) | 62 | 0.00E+00 | 86.08 |
| *FBGS/+; UAS chinmo^RNAi^/+ AL-RU* | 72(97) | 42 |  |  |
| *FBGS/+; UAS chinmo^RNAi^/+ DR+RU* | 102(88) | 60 | 7.80E-07 | 24.41 |
| *FBGS/+; UAS chinmo^RNAi^/+ AL+RU* | 80(98) | 50 |  |  |
|  | | | | |
| Experiment 2 |  |  |  |  |
| *FBGS/+; UAS chinmo^RNAi^/+ DR-RU* | 96(205) | 42 | 1.00E-05 | 19.5 |
| *FBGS/+; UAS chinmo^RNAi^/+ DR +RU* | 98(179) | 54 |  |  |
| *FBGS/+; UAS chinmo^RNAi^/+ AL-RU* | 58(182) | 30 | 0.00E+00 | 49.65 |
| *FBGS/+; UAS chinmo^RNAi^/+ AL+RU* | 70(178) | 40 |  |  |
| *FBGS/+; UAS chinmo^RNAi^/+ DR-RU* | 96(205) | 42 | 0.00E+00 | 104.64 |
| *FBGS/+; UAS chinmo^RNAi^/+ AL-RU* | 58(182) | 30 |  |  |
| *FBGS/+; UAS chinmo^RNAi^/+ DR+RU* | 98(179) | 54 | 0.00E+00 | 136.78 |
| *FBGS/+; UAS chinmo^RNAi^/+ AL+RU* | 70(178) | 40 |  |  |

^#^Experiment 1 is represented in Figure 3; *p value calculated by log rank test; *** **χ^2^, Chi^2^ calculated by Log rank test.

**Figure 3-source data 1B.** Cox proportional of *FBGS> UAS chinmo^RNAi^* strain.

|  | **Risk factor** | **p value** |
| --- | --- | --- |
| Experiment 1^#^  *FBGS/+; UAS chinmo^RNAi^/+* | Diet | 0.000207 |
|  | Ligand | 0.069844 |
| Experiment 2  *FBGS/+; UAS chinmo^RNAi^/+* | Diet | 0.000001 |
|  | Ligand | 0.154592 |
