# Supplementary material for: miR-125-chinmo pathway regulates dietary restriction-dependent enhancement of lifespan in Drosophila
Source: eLife. 2021 Jun 8;10:e62621. doi: 10.7554/eLife.62621 (PMC8233039; doi:10.7554/eLife.62621)
Supplement: Figure 4—source data 5. [file elife-62621-fig4-data5.docx]

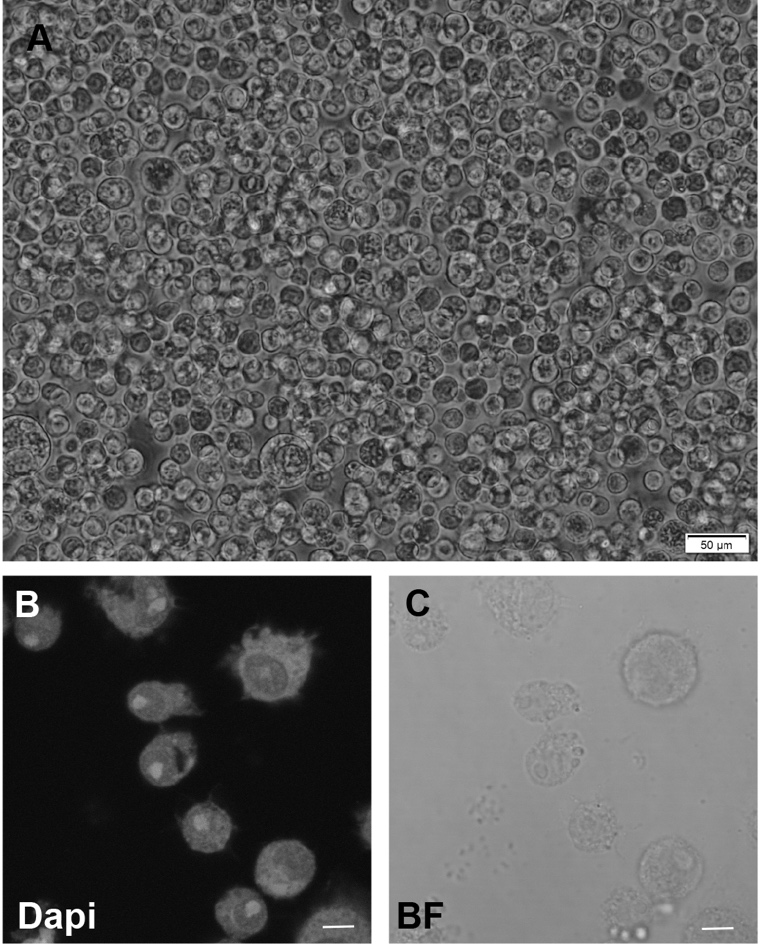


**Figure 4-source data 5. Morphology and fluorescence microscopy of Kc167 cells used in Figure 4. (A)** Bright field image of the cultured Kc167 cells used for characterization of Chinmo mutant constructs. (B) Fluorescent microscopy of fixed Kc167 cells stained with Dapi. No specks of fluorescent signal were obtained in spaces surrounding the cell indicating that Mycoplasma contamination was absent from the culture.
