# Supplementary material for: miR-125-chinmo pathway regulates dietary restriction-dependent enhancement of lifespan in Drosophila
Source: eLife. 2021 Jun 8;10:e62621. doi: 10.7554/eLife.62621 (PMC8233039; doi:10.7554/eLife.62621)
Supplement: Figure 5—source data 1. [file elife-62621-fig5-data1.docx]

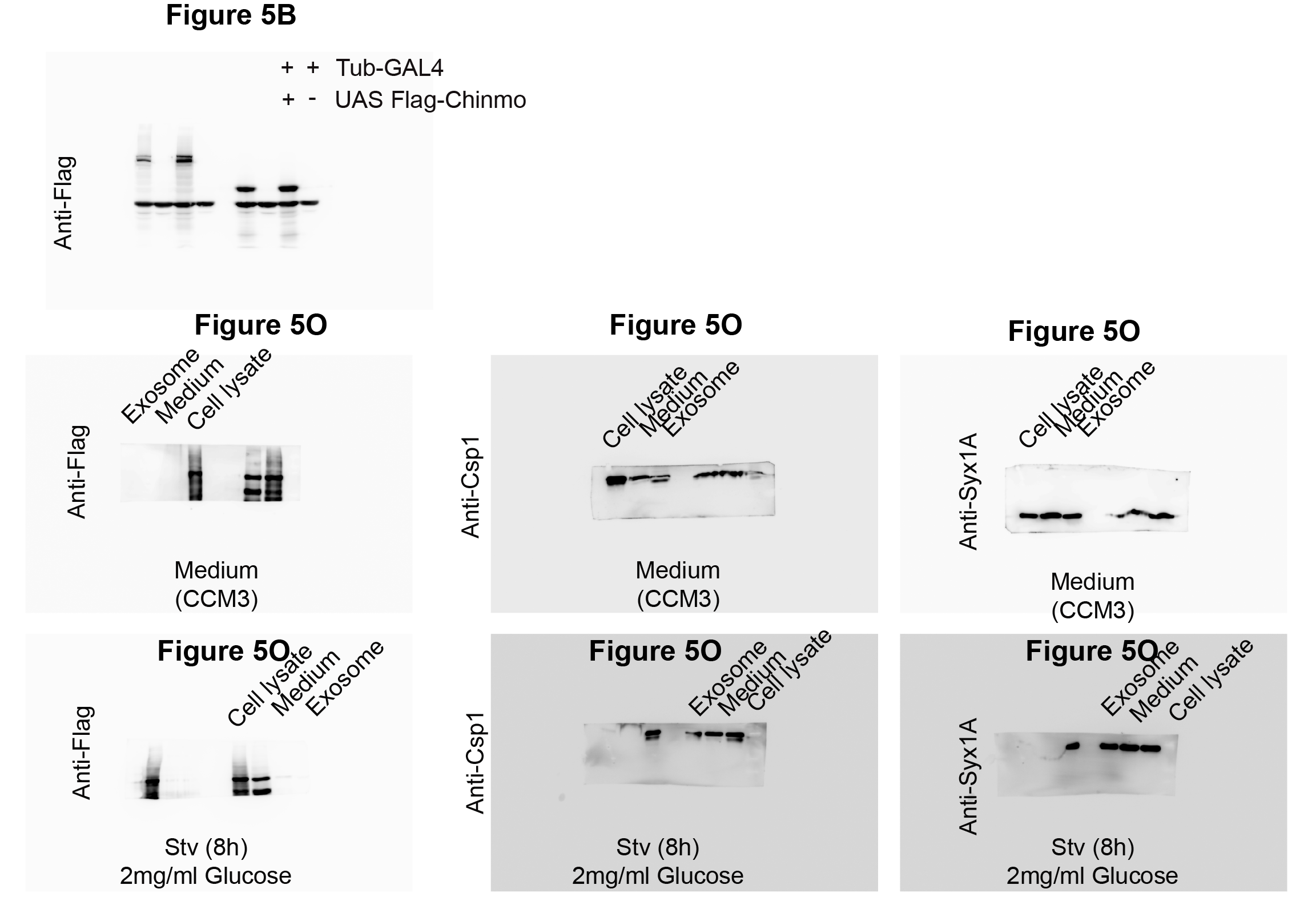


**Figure 5-figure -source data 1. Uncropped western blots with relevant bands labeled for Figure 5B and 5O.**
