# Supplementary figures and images for: miR-125-chinmo pathway regulates dietary restriction-dependent enhancement of lifespan in Drosophila
Source: eLife. 2021 Jun 8;10:e62621. doi: 10.7554/eLife.62621 (PMC8233039; doi:10.7554/eLife.62621)

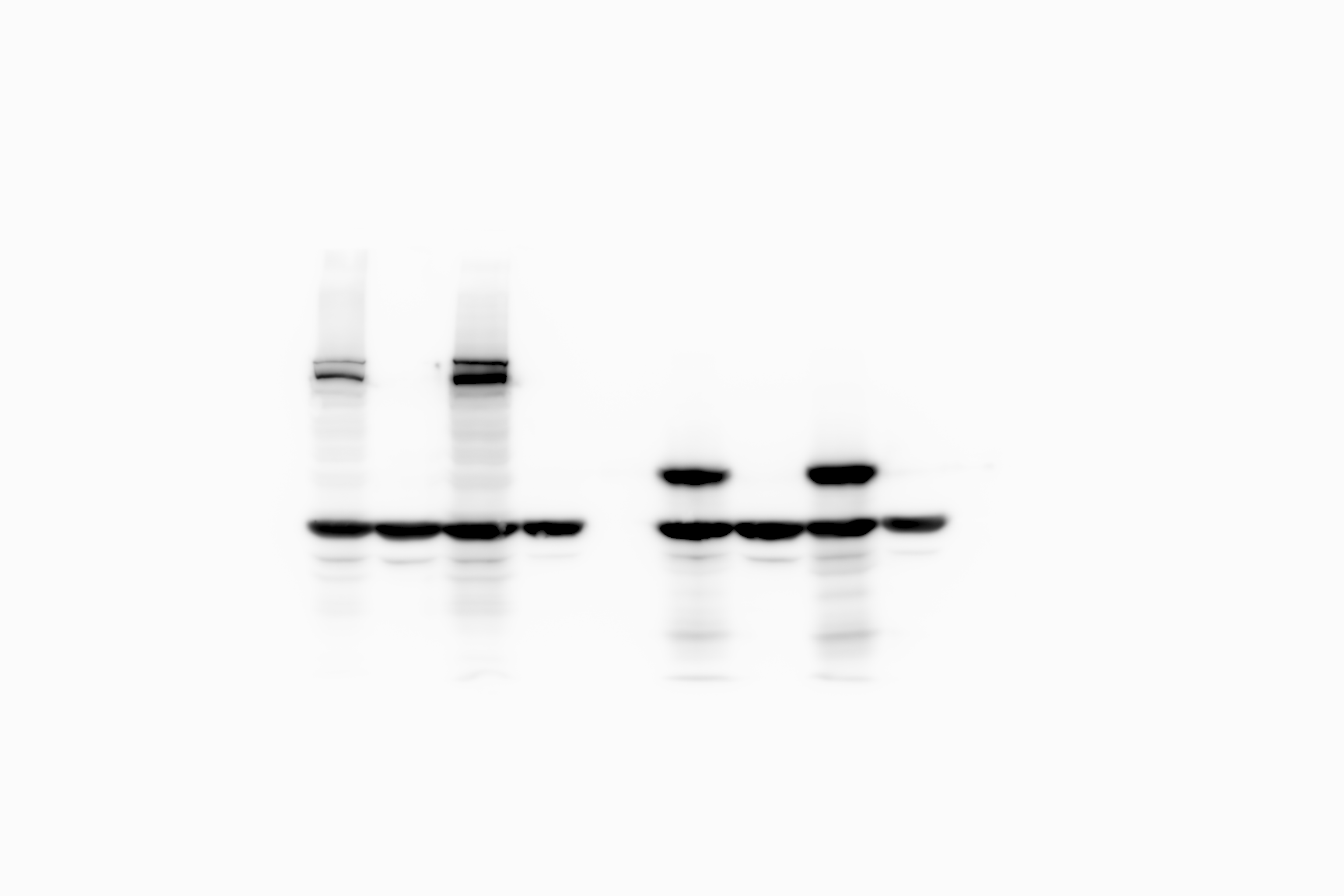

Supplement: Figure 5—source data 2. [file elife-62621-fig5-data2.zip › Figure 5-source data 2/Figure 5 B Flag Chinmo and Tubulin 20 sec.tif]

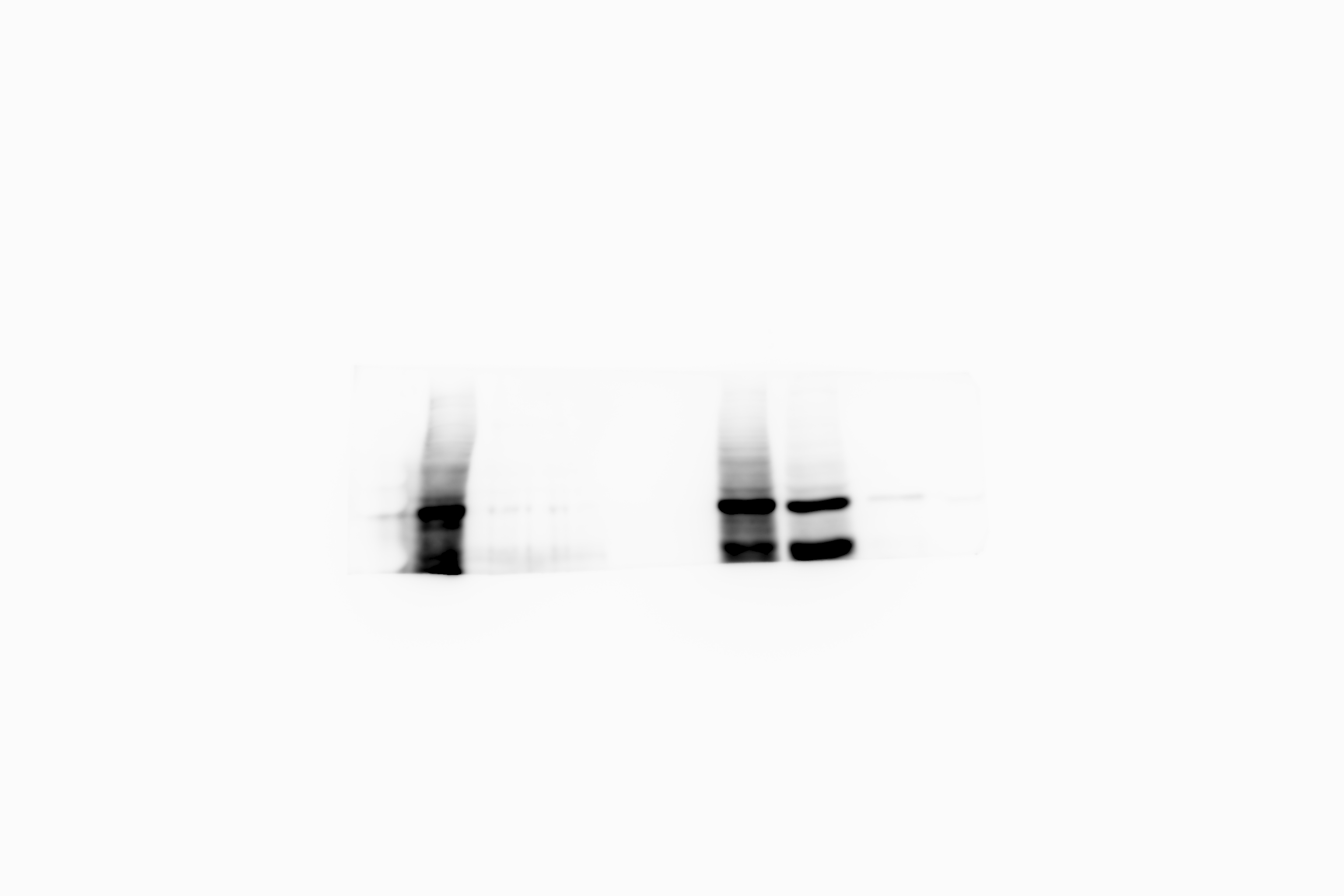

Supplement: Figure 5—source data 2. [file elife-62621-fig5-data2.zip › Figure 5-source data 2/Figure 5 O starvation Flag chinmo 1 min.tif]

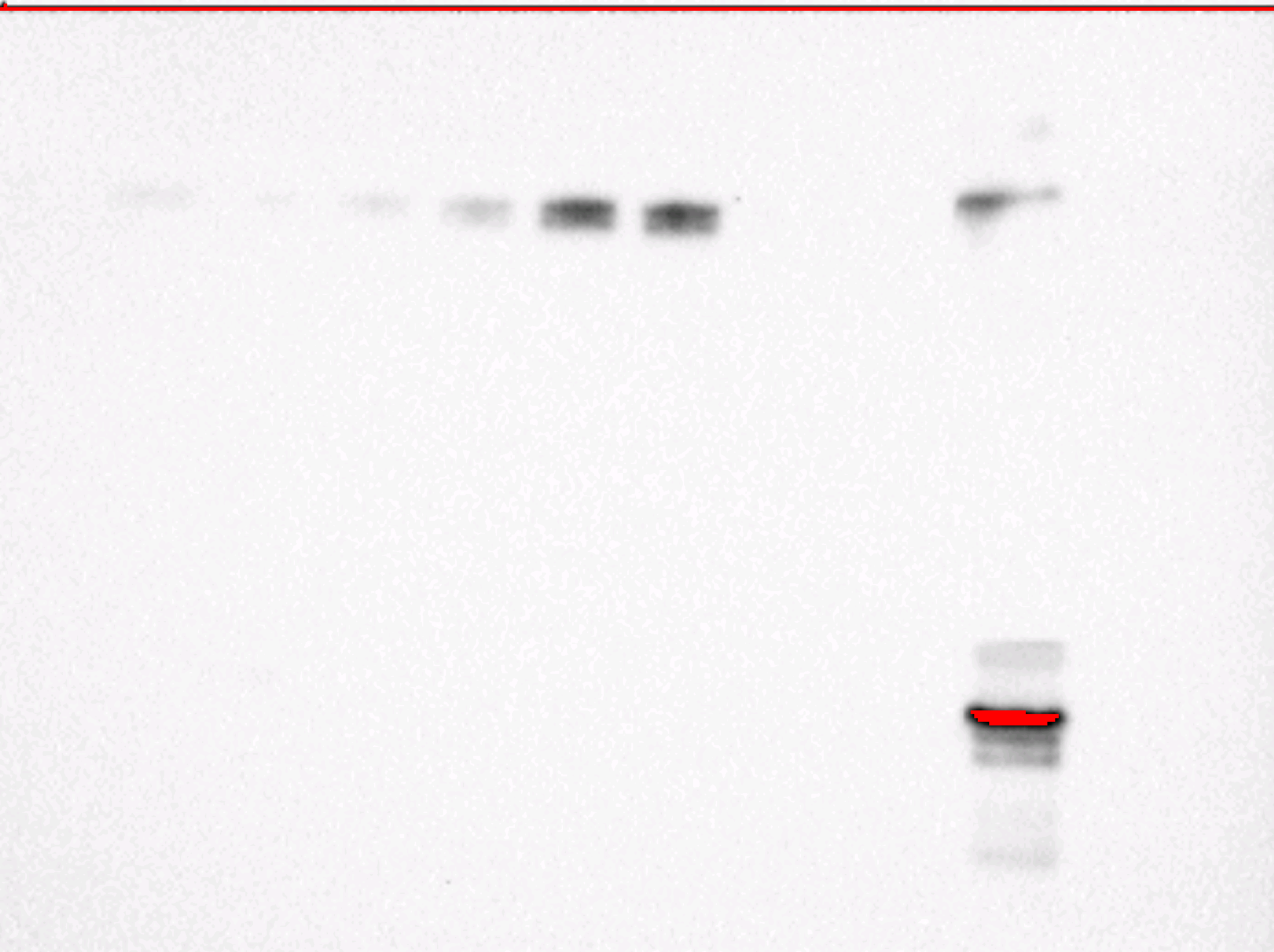

Supplement: Figure 5—figure supplement 1—source data 2. [file elife-62621-fig5-figsupp1-data2.zip › Figure 5-figure supplement 1-source data 2/Figure 5 figure supplement 1 Csp1 gc 6-21-2020 pico chemi 1a 2020-06-21 19hr 01min_Exposure_22.0sec.tif]

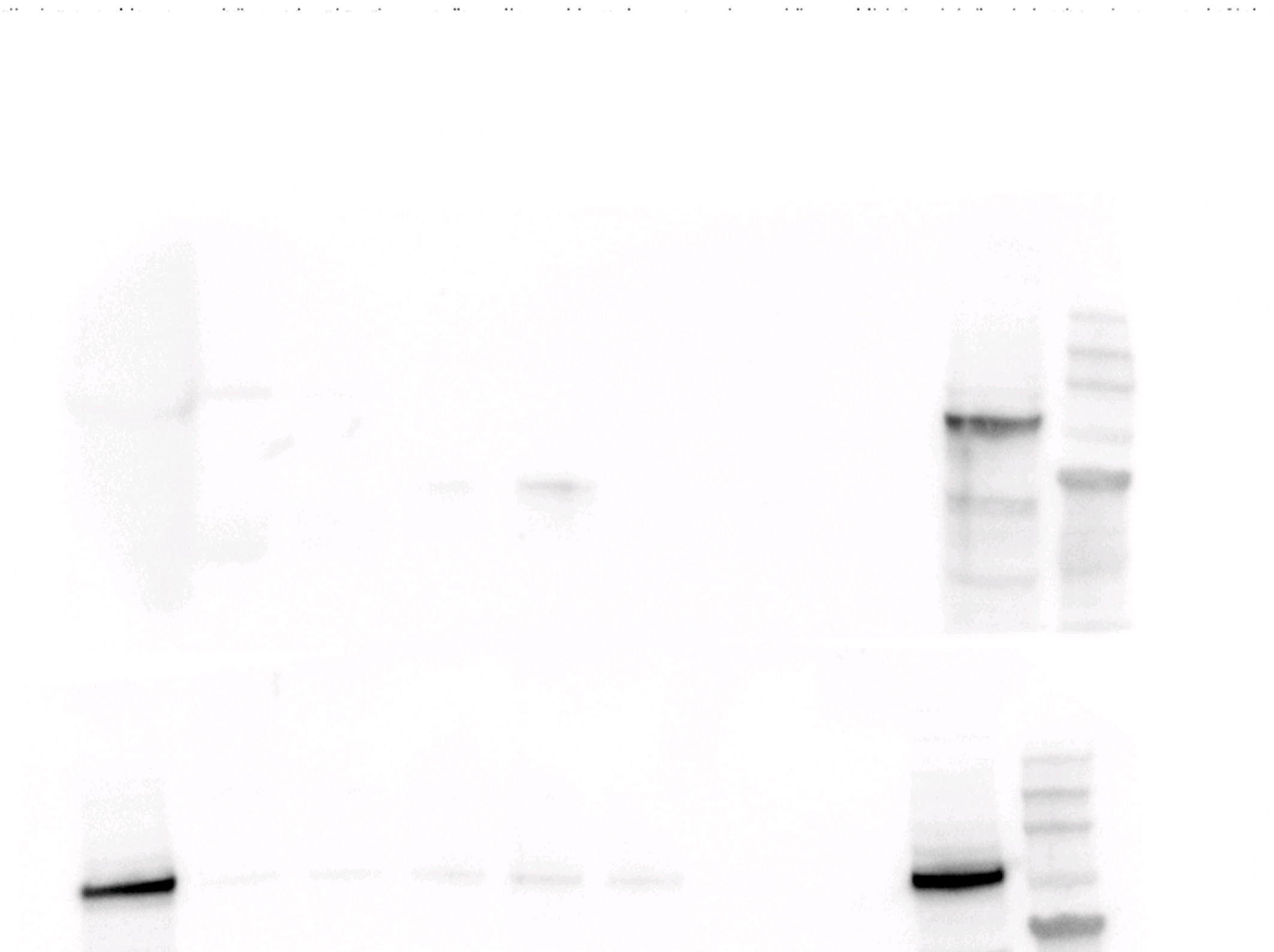

Supplement: Figure 5—figure supplement 1—source data 2. [file elife-62621-fig5-figsupp1-data2.zip › Figure 5-figure supplement 1-source data 2/Figure 5 figure supplement 1 Hrs FL gc6-25-2020 chemi4B 2020-06-25 16hr 49min_Exposure_1.0sec.tif]

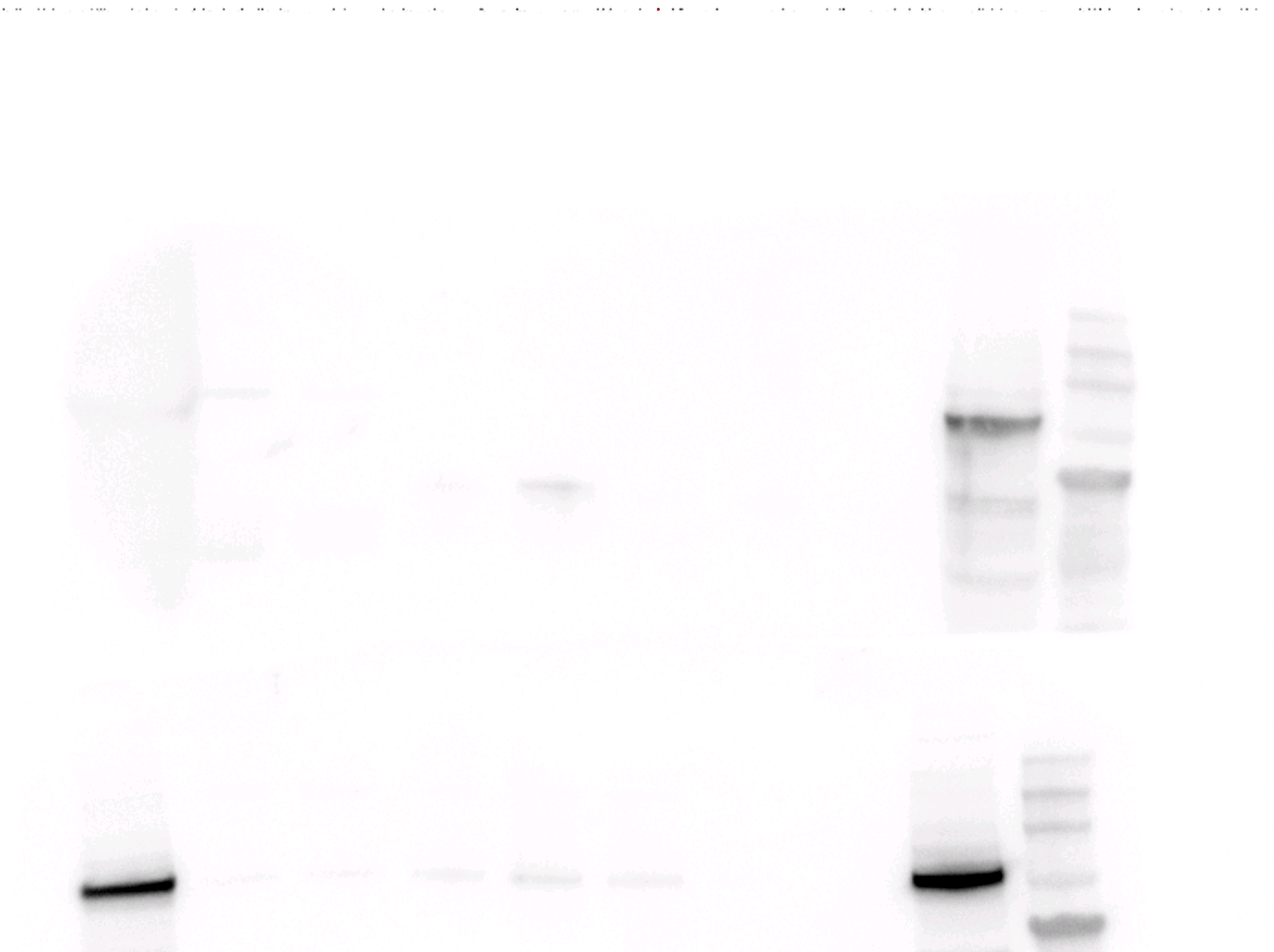

Supplement: Figure 5—figure supplement 1—source data 2. [file elife-62621-fig5-figsupp1-data2.zip › Figure 5-figure supplement 1-source data 2/Figure 5 syntaxin figure supplement 1 gc6-25-2020 chemi4A 2020-06-25 16hr 49min_Exposure_1.0sec.tif]
