# Supplementary material for: miR-125-chinmo pathway regulates dietary restriction-dependent enhancement of lifespan in Drosophila
Source: eLife. 2021 Jun 8;10:e62621. doi: 10.7554/eLife.62621 (PMC8233039; doi:10.7554/eLife.62621)
Supplement: Figure 6—source data 1. [file elife-62621-fig6-data1.docx]

**Figure 6-source data 1A.** Lifespan analysis of strains used in Figure 6.

|  | **Lifespan (Days)** | | **p value** | **χ^2^** |
| --- | --- | --- | --- | --- |
| Genotype | Maximum  (Number of flies) | Median |  |  |
| Experiment 1* |  |  |  |  |
| *ElavGS/+; ElavGS/+; UAS chinmo/ElavGS AL-RU* | 56(98) | 32 | 0.00E+00 | 70.08 |
| *ElavGS/+; ElavGS/+; UAS chinmo/ElavGS DR -RU* | 88(99) | 42 |  |  |
| *ElavGS/+; ElavGS/+; UAS chinmo/ElavGS AL +RU* | 50(100) | 10 | 0.0066 | 7.37 |
| *ElavGS/+; ElavGS/+; UAS chinmo/ElavGS DR+RU* | 52(95) | 16 |  |  |
| *ElavGS/+; ElavGS/+; UAS chinmo/ElavGS AL -RU* | 56(98) | 32 | 0.00E+00 | 44.56 |
| *ElavGS/+; ElavGS/+; UAS chinmo/ElavGS AL +RU* | 50(100) | 10 |  |  |
| *ElavGS/+; ElavGS/+; UAS chinmo/ElavGS DR -RU* | 88(99) | 42 | 0.00E+00 | 139.2 |
| *ElavGS/+; ElavGS/+; UAS chinmo/ElavGS DR+RU* | 52(95) | 16 |  |  |
| Experiment 2 |  |  |  |  |
| *ElavGS/+; ElavGS/+; UAS chinmo/ElavGS AL-RU* | 58(242) | 28 | 0.00E+00 | 173.22 |
| *ElavGS/+; ElavGS/+; UAS chinmo/ElavGS DR -RU* | 80(168) | 44 |  |  |
| *ElavGS/+; ElavGS/+; UAS chinmo/ElavGS AL +RU* | 54(216) | 12 | 1.30E-05 | 18.95 |
| *ElavGS/+; ElavGS/+; UAS chinmo/ElavGS DR+RU* | 58(179) | 16 |  |  |
| *ElavGS/+; ElavGS/+; UAS chinmo/ElavGS AL -RU* | 58(242) | 28 | 0.00E+00 | 48.54 |
| *ElavGS/+; ElavGS/+; UAS chinmo/ElavGS AL +RU* | 54(216) | 12 |  |  |
| *ElavGS/+; ElavGS/+; UAS chinmo/ElavGS DR -RU* | 80(168) | 44 | 0.00E+00 | 150.29 |
| *ElavGS/+; ElavGS/+; UAS chinmo/ElavGS DR+RU* | 58(179) | 16 |  |  |
| Experiment 1** |  |  |  |  |
| *FBGS/+; UAS chinmo/+ AL-RU* | 64(186) | 26 | 0.00E+00 | 144.82 |
| *FBGS/+; UAS chinmo/+ DR-RU* | 80(187) | 52 |  |  |
| *FBGS/+; UAS chinmo/+ AL+RU* | 32(213) | 16 | 0.0283 | 4.81 |
| *FBGS/+; UAS chinmo/+ DR+RU* | 42(209) | 16 |  |  |
| *FBGS/+; UAS chinmo/+ AL-RU* | 64(186) | 26 | 0.00E+00 | 160.28 |
| *FBGS/+; UAS chinmo/+ AL+RU* | 32(213) | 16 |  |  |
| *FBGS/+; UAS chinmo/+ DR-RU* | 80(187) | 52 | 0.00E+00 | 282.69 |
| *FBGS/+; UAS chinmo/+ DR+RU* | 42(209) | 16 |  |  |
| Experiment 2 |  |  |  |  |
| *FBGS/+; UAS chinmo/+ AL-RU* | 38(78) | 30 | 0.00E+00 | 55.81 |
| *FBGS/+; UAS chinmo/+ DR-RU* | 68(80) | 40 |  |  |
| *FBGS/+; UAS chinmo/+ AL+RU* | 36(131) | 16 | 1.20E-05 | 19.22 |
| *FBGS/+; UAS chinmo/+ DR+RU* | 38(138) | 20 |  |  |
| *FBGS/+; UAS chinmo/+ AL-RU* | 38(78) | 30 | 0.00E+00 | 59.14 |
| *FBGS/+; UAS chinmo/+ AL+RU* | 36(131) | 16 |  |  |
| *FBGS/+; UAS chinmo/+ DR-RU* | 68(80) | 40 | 0.00E+00 | 126.46 |
| *FBGS/+; UAS chinmo/+ DR+RU* | 38(138) | 20 |  |  |
| Experiment 1*** |  |  |  |  |
| *ElavGS/+; ElavGS/+; UAS Flag chinmo/ElavGS AL-RU* | 74(107) | 44 | 0.00E+00 | 108.8 |
| *ElavGS/+; ElavGS/+; UAS Flag chinmo/ElavGS DR -RU* | 104(99) | 70 |  |  |
| *ElavGS/+; ElavGS/+; UAS Flag chinmo/ElavGS AL +RU* | 54(137) | 18 | 0.0068 | 7.34 |
| *ElavGS/+; ElavGS/+; UAS Flag chinmo/ElavGS DR+RU* | 54(100) | 26 |  |  |
| *ElavGS/+; ElavGS/+; UAS Flag chinmo/ElavGS AL -RU* | 74(107) | 44 | 0.00E+00 | 83.14 |
| *ElavGS/+; ElavGS/+; UAS Flag chinmo/ElavGS AL +RU* | 54(137) | 18 |  |  |
| *ElavGS/+; ElavGS/+; UAS Flag chinmo/ElavGS DR -RU* | 104(99) | 70 | 0.00E+00 | 203.1 |
| *ElavGS/+; ElavGS/+; UAS Flag chinmo/ElavGS DR+RU* | 54(100) | 26 |  |  |
| Experiment 2 |  |  |  |  |
| *ElavGS/+; ElavGS/+; UAS Flag chinmo/ElavGS AL -RU* | 76(143) | 42 | 0.00E+00 | 64.59 |
| *ElavGS/+; ElavGS/+; UAS Flag chinmo/ElavGS DR -RU* | 104(102) | 59 |  |  |
| *ElavGS/+; ElavGS/+; UAS Flag chinmo/ElavGS AL +RU* | 60(114) | 18 | 0.2506 | 1.32 |
| *ElavGS/+; ElavGS/+; UAS Flag chinmo/ElavGS DR+RU* | 66(95) | 20 |  |  |
| *ElavGS/+; ElavGS/+; UAS Flag chinmo/ElavGS AL -RU* | 76(143) | 42 | 0.00E+00 | 100.85 |
| *ElavGS/+; ElavGS/+; UAS Flag chinmo/ElavGS AL +RU* | 60(114) | 18 |  |  |
| *ElavGS/+; ElavGS/+; UAS Flag chinmo/ElavGS DR -RU* | 104(102) | 59 | 0.00E+00 | 109.5 |
| *ElavGS/+; ElavGS/+; UAS Flag chinmo/ElavGS DR+RU* | 66(95) | 20 |  |  |
| Experiment 1 |  |  |  |  |
| *FBGS/+; UAS Flag chinmo/+ AL-RU* | 48(72) | 29 | 5.10E-06 | 20.79 |
| *FBGS/+; UAS Flag chinmo/+DR-RU* | 58(100) | 40 |  |  |
| *FBGS/+; UAS Flag chinmo/+AL+RU* | 26(92) | 13 | 0.0546 | 3.695 |
| *FBGS/+; UAS Flag chinmo/+DR+RU* | 26(72) | 16 |  |  |
| *FBGS/+; UAS Flag chinmo/+AL-RU* | 48(72) | 29 | 0.00E+00 | 140.5 |
| *FBGS/+; UAS Flag chinmo/+AL+RU* | 26(92) | 13 |  |  |
| *FBGS/+; UAS Flag chinmo/+DR-RU* | 58(100) | 40 | 0.00E+00 | 196.9 |
| *FBGS/+; UAS Flag chinmo/+DR+RU* | 26(72) | 16 |  |  |
| Experiment 2**** |  |  |  |  |
| *FBGS/+; UAS Flag chinmo/+AL-RU* | 70(96) | 42 | 0.00E+00 | 37.76 |
| *FBGS/+; UAS Flag chinmo/+DR-RU* | 80(91) | 54 |  |  |
| *FBGS/+; UAS Flag chinmo/+AL+RU* | 30(83) | 14 | 0.9472 | 0.004379 |
| *FBGS/+; UAS Flag chinmo/+DR+RU* | 28(73) | 14 |  |  |
| *FBGS/+; UAS Flag chinmo/+AL-RU* | 70(96) | 42 | 0.00E+00 | 178.5 |
| *FBGS/+; UAS Flag chinmo/+AL+RU* | 30(83) | 14 |  |  |
| *FBGS/+; UAS Flag chinmo/+DR-RU* | 80(91) | 54 | 0.00E+00 | 175 |
| *FBGS/+; UAS Flag chinmo/+DR+RU* | 28(73) | 14 |  |  |

^*^Experiment 1 is represented in Figure 6B; ^**^Experiment 1 is represented in Figure 6C; ^***^Experiment 1 is represented in Figure 6D; ^****^Experiment 2 is represented in Figure 6E; p value calculated by log rank test; χ^2^, Chi^2^ calculated by Log rank test.

**Figure 6-source data 1B.** Cox proportional of strains used in Figure 6.

| **Genotype (Experiment)** | **Risk factor** | **p value** |
| --- | --- | --- |
| *ElavGS/+; ElavGS/+; UAS chinmo/ElavGS (*Experiment 1*) | Diet | 0.019764 |
|  | Ligand | 0.00272 |
| *ElavGS/+; ElavGS/+; UAS chinmo/ElavGS (*Experiment 2) | Diet | 0.010629 |
|  | Ligand | 0.010629 |
| *FBGS/+; UAS chinmo/+* (Experiment 1) | Diet | 0.0098 |
|  | Ligand | 0 |
| *FBGS/+; UAS chinmo/+* (Experiment 2) | Diet | 0.005625 |
|  | Ligand | 0.005625 |
| *ElavGS/+; ElavGS/+; UAS Flag chinmo/ElavGS (*Experiment 1^#^) | Diet | 0.011665 |
|  | Ligand | 0.000001 |
| *ElavGS/+; ElavGS/+; UAS Flag chinmo/ElavGS (*Experiment 2) | Diet | 0.005342 |
|  | Ligand | 0.000012 |
| *FBGS/+; UAS Flag chinmo/+* (Experiment 1) | Diet | 0.172085 |
|  | Ligand | 0.000007 |
| *FBGS/+; UAS Flag chinmo/+* (Experiment 2) | Diet | 0.238178 |
|  | Ligand | 0 |
