# Supplementary material for: miR-125-chinmo pathway regulates dietary restriction-dependent enhancement of lifespan in Drosophila
Source: eLife. 2021 Jun 8;10:e62621. doi: 10.7554/eLife.62621 (PMC8233039; doi:10.7554/eLife.62621)
Supplement: Figure 6—figure supplement 1—source data 1. [file elife-62621-fig6-figsupp1-data1.docx]

**Figure 6-figure supplement 1-source data 1A.** Lifespan analysis to test the effect of genetic background and RU-486 on life span of strains used in Figure 6.

| **Genotype** | **Lifespan (Days)** | | **p value** | **χ^2^** |
| --- | --- | --- | --- | --- |
| Experiment 1^#^ | Maximum  (Number of flies) | Median |  |  |
| *ElavGS/+; ElavGS/+; ElavGS/+ AL-RU* | 61(194) | 33 | 0.00E+00 | 295.1 |
| *ElavGS/+; ElavGS/+; ElavGS/+ DR-RU* | 77(176) | 65 |  |  |
| *ElavGS/+; ElavGS/+; ElavGS/+ AL+RU* | 63(189) | 40 | 0.00E+00 | 259.61 |
| *ElavGS/+; ElavGS/+; ElavGS/+ DR+RU* | 75(187) | 61 |  |  |
| *ElavGS/+; ElavGS/+; ElavGS/+ AL-RU* | 61(194) | 33 | 0.0002 | 13.52 |
| *ElavGS/+; ElavGS/+; ElavGS/+ AL+RU* | 63(189) | 40 |  |  |
| *ElavGS/+; ElavGS/+; ElavGS/+ DR-RU* | 77(176) | 65 | 0.0003 | 13.35 |
| *ElavGS/+; ElavGS/+; ElavGS/+ DR+RU* | 75(187) | 61 |  |  |
| Experiment 2 |  |  |  |  |
| *ElavGS/+; ElavGS/+; ElavGS/+ AL-RU* | 33(194) | 23 | 0.00E+00 | 392.9 |
| *ElavGS/+; ElavGS/+; ElavGS/+ DR-RU* | 77(186) | 63 |  |  |
| *ElavGS/+; ElavGS/+; ElavGS/+ AL+RU* | 36(194) | 26 | 0.00E+00 | 384.87 |
| *ElavGS/+; ElavGS/+; ElavGS/+ DR+RU* | 75(187) | 61 |  |  |
| *ElavGS/+; ElavGS/+; ElavGS/+ AL-RU* | 33(194) | 23 | 0.0081 | 7.005 |
| *ElavGS/+; ElavGS/+; ElavGS/+ AL+RU* | 36(194) | 26 |  |  |
| *ElavGS/+; ElavGS/+; ElavGS/+ DR-RU* | 77(186) | 63 | 0.0036 | 8.485 |
| *ElavGS/+; ElavGS/+; ElavGS/+ DR+RU* | 75(187) | 61 |  |  |
| Experiment 1^##^ |  |  |  |  |
| *+/+; UAS chinmo/+ AL -RU* | 40(79) | 24 | 0.00E+00 | 62.24 |
| *+/+; UAS chinmo/+ DR -RU* | 72(119) | 34 |  |  |
| *+/+; UAS chinmo/+ AL +RU* | 34(92) | 20 | 0.00E+00 | 90.04 |
| *+/+; UAS chinmo/+ DR+RU* | 68(116) | 38 |  |  |
| *+/+; UAS chinmo/+ AL -RU* | 40(79) | 24 | 0.0059 | 7.581 |
| *+/+; UAS chinmo/+ AL +RU* | 34(92) | 20 |  |  |
| *+/+; UAS chinmo/+ DR -RU* | 72(119) | 34 | 0.2500 | 1.324 |
| *+/+; UAS chinmo/+ DR+RU* | 68(116) | 38 |  |  |
| Experiment 1^###^ |  |  |  |  |
| *+/+; UAS Flag chinmo/+ AL -RU* | 50(98) | 26 | 0.00E+00 | 42.33 |
| *+/+; UAS Flag chinmo/+ DR -RU* | 72(103) | 38 |  |  |
| *+/+; UAS Flag chinmo/+ AL +RU* | 44(85) | 30 | 0.00E+00 | 50.27 |
| *+/+; UAS Flag chinmo/+ DR+RU* | 70(80) | 38 |  |  |
| *+/+; UAS Flag chinmo/+ AL -RU* | 50(98) | 26 | 0.9784 | 0.0006823 |
| *+/+; UAS Flag chinmo/+ AL +RU* | 44(85) | 30 |  |  |
| *+/+; UAS Flag chinmo/+ DR -RU* | 72(103) | 38 | 0.6929 | 0.1559 |
| *+/+; UAS Flag chinmo/+ DR+RU* | 70(80) | 38 |  |  |
| Experiment 2 |  |  |  |  |
| *+/+; UAS Flag chinmo/+ AL -RU* | 44(126) | 30 | 0.00E+00 | 233.5 |
| *+/+; UAS Flag chinmo/+ DR -RU* | 70(120) | 54 |  |  |
| *+/+; UAS Flag chinmo/+ AL +RU* | 60(126) | 36 | 0.00E+00 | 205.4 |
| *+/+; UAS Flag chinmo/+ DR+RU* | 70(116) | 56 |  |  |
| *+/+; UAS Flag chinmo/+ AL -RU* | 44(126) | 30 | 3.30E-06 | 21.61 |
| *+/+; UAS Flag chinmo/+ AL +RU* | 60(126) | 36 |  |  |
| *+/+; UAS Flag chinmo/+ DR -RU* | 70(120) | 54 | 0.336 | 0.9268 |
| *+/+; UAS Flag chinmo/+ DR+RU* | 70(116) | 56 |  |  |

^#^Experiment 1 is represented in Figure 6-figure supplement 1A; ^##^Experiment 1 is represented in Figure 6-figure supplement 1B; ^###^Experiment 1 is represented in Figure 6-figure supplement 1C; p value calculated by log rank test; χ^2^, Chi^2^ calculated by Log rank test.

**Figure 6-figure supplement 1-source data 1B.** Cox proportional hazards regression analysis to test the effect of genetic background RU-486 for strains used in Figure 6.

| **Genotype (Experiment)** | **Risk factor** | **p value** |
| --- | --- | --- |
| *ElavGS/+; ElavGS/+; ElavGS/+* (Experiment 1) | Diet | 0.007065 |
|  | Ligand (RU-486) | 0.87033 |
| *ElavGS/+; ElavGS/+; ElavGS/+* (Experiment 2) | Diet | 0 |
|  | Ligand (RU-486) | 0.824668 |
| *+/+; UAS chinmo/+* (Experiment 1) | Diet | 0 |
|  | Ligand (RU-486) | 0.2562 |
| *+/+; UAS Flag chinmo/+* (Experiment 1) | Diet | 0.000025 |
|  | Ligand (RU-486) | 0.392227 |
| *+/+; UAS Flag chinmo/+* (Experiment 2) | Diet | 0.001015 |
|  | Ligand (RU-486) | 0.227363 |
