# Supplementary material for: miR-125-chinmo pathway regulates dietary restriction-dependent enhancement of lifespan in Drosophila
Source: eLife. 2021 Jun 8;10:e62621. doi: 10.7554/eLife.62621 (PMC8233039; doi:10.7554/eLife.62621)
Supplement: Figure 7—source data 1. [file elife-62621-fig7-data1.docx]

**Figure 7- source data 1A.** Lifespan analysis of *FBGS> UAS fasn1^RNAi^*.

|  | **Lifespan (Days)** | | **p value** | **χ^2^** |
| --- | --- | --- | --- | --- |
| Experiment 1 | Maximum (Number of flies) | Median |  |  |
| *FBGS/+; UAS FASN1^RNAi^/+ AL -RU* | 54(188) | 32 | 0.00E+00 | 69.60 |
| *FBGS/+; UAS FASN1^RNAi^/+ DR -RU* | 98(136) | 42 |  |  |
| *FBGS/+; UAS FASN1^RNAi^/+ AL +RU* | 44(123) | 30 | 0.00E+00 | 65.62 |
| *FBGS/+; UAS FASN1^RNAi^/+ DR +RU* | 86(127) | 40 |  |  |
| *FBGS/+; UAS FASN1^RNAi^/+ AL -RU* | 54(188) | 32 | 8.30E-06 | 19.86 |
| *FBGS/+; UAS FASN1^RNAi^/+ AL +RU* | 44(123) | 30 |  |  |
| *FBGS/+; UAS FASN1^RNAi^/+ DR -RU* | 98(136) | 42 | 0.0334 | 4.525 |
| *FBGS/+; UAS FASN1^RNAi^/+ DR +RU* | 86(127) | 30 |  |  |
| ^#^Experiment 2 |  |  |  |  |
| *FBGS/+; UAS FASN1^RNAi^/+ AL -RU* | 62(124) | 32 | 0.00E+00 | 115.2 |
| *FBGS/+; UAS FASN1^RNAi^/+ DR -RU* | 104(100) | 62 |  |  |
| *FBGS/+; UAS FASN1^RNAi^/+ AL +RU* | 44(106) | 26 | 0.00E+00 | 167.5 |
| *FBGS/+; UAS FASN1^RNAi^/+ DR +RU* | 86(110) | 60 |  |  |
| *FBGS/+; UAS FASN1^RNAi^/+ AL -RU* | 62(124) | 32 | 6.70E-06 | 20.26 |
| *FBGS/+; UAS FASN1^RNAi^/+ AL +RU* | 44(106) | 26 |  |  |
| *FBGS/+; UAS FASN1^RNAi^/+ DR -RU* | 104(100) | 62 | 0.0156 | 5.850 |
| *FBGS/+; UAS FASN1^RNAi^/+ DR +RU* | 86(110) | 60 |  |  |

^#^Experiment 2 is represented in Figure 7I; p value calculated by log rank test; χ^2^, Chi^2^ calculated by Log rank test.

**Figure 7- source data 1B.** Cox regression analysis of *FBGS> UAS fasn1^RNAi^*.

|  | **Risk factor** | **p value** |
| --- | --- | --- |
| Experiment 1 | Diet | 0 |
|  | Ligand | 0.03420 |
| Experiment 2^#^ | Diet | 0 |
|  | Ligand | 0.0023 |

**Figure 7- source data 1C.** Lifespan analysis of *+/+; UAS fasn1^RNAi^/+* genotype.

|  | **Lifespan (Days)** | | **p value** | **χ^2^** |
| --- | --- | --- | --- | --- |
| Experiment | Maximum (Number of flies) | Median |  |  |
| *+/+; UAS FASN1^RNAi^/+ AL -RU* | 38(108) | 28 | 0.00E+00 | 137.3 |
| *+/+; UAS FASN1^RNAi^/+ DR -RU* | 72(110) | 50 |  |  |
| *+/+; UAS FASN1^RNAi^/+ AL +RU* | 36(106) | 26 | 0.00E+00 | 151.58 |
| *+/+; UAS FASN1^RNAi^/+ DR +RU* | 76(90) | 46 |  |  |
| *+/+; UAS FASN1^RNAi^/+ AL -RU* | 38(108) | 28 | 0.8532 | 0.03 |
| *+/+; UAS FASN1^RNAi^/+ AL +RU* | 36(106) | 26 |  |  |
| *+/+; UAS FASN1^RNAi^/+ DR -RU* | 72(110) | 50 | 0.2536 | 1.3 |
| *+/+; UAS FASN1^RNAi^/+ DR +RU* | 76(90) | 46 |  |  |

^#^Experiment is represented in Figure 7-figure supplement 3A.

**Figure 7- source data 1D.** Cox regression analysis of *+/+; UAS fasn1^RNAi^/+* genotype.

|  | **Risk factor** | **p value** |
| --- | --- | --- |
| Experiment 1 | Diet | 0 |
|  | Ligand | 0.6213 |
