# Supplementary material for: miR-125-chinmo pathway regulates dietary restriction-dependent enhancement of lifespan in Drosophila
Source: eLife. 2021 Jun 8;10:e62621. doi: 10.7554/eLife.62621 (PMC8233039; doi:10.7554/eLife.62621)
Supplement: Figure 7—source data 2. [file elife-62621-fig7-data2.docx]

**Figure 7-source data 2A.** Lifespan analysis of *FBGS> UAS fatp^RNAi^*.

|  | **Lifespan (Days)** | | **p value** | **χ^2^** |
| --- | --- | --- | --- | --- |
| Experiment 1 | Maximum (Number of flies) | Median |  |  |
| *FBGS/+; UAS fatp^RNAi^/+ AL -RU* | 62(123) | 34 | 0.00E+00 | 40.74 |
| *FBGS/+; UAS fatp^RNAi^/+ DR -RU* | 94(97) | 46 |  |  |
| *FBGS/+; UAS fatp^RNAi^/+ AL +RU* | 58(113) | 36 | 2.10E-05 | 18.11 |
| *FBGS/+; UAS fatp^RNAi^/+ DR +RU* | 74(124) | 40 |  |  |
| *FBGS/+; UAS fatp^RNAi^/+ AL -RU* | 62(123) | 34 | 0.9425 | 0.01 |
| *FBGS/+; UAS fatp^RNAi^/+ AL +RU* | 58(113) | 36 |  |  |
| *FBGS/+; UAS fatp^RNAi^/+ DR -RU* | 94(97) | 46 | 3.00E-05 | 17.41 |
| *FBGS/+; UAS fatp^RNAi^/+ DR +RU* | 74(124) | 40 |  |  |
| ^#^Experiment 2 |  |  |  |  |
| *FBGS/+; UAS fatp^RNAi^/+ AL -RU* | 58(85) | 36 | 0.00E+00 | 70.01 |
| *FBGS/+; UAS fatp^RNAi^/+ DR -RU* | 96(79) | 54 |  |  |
| *FBGS/+; UAS fatp^RNAi^/+ AL +RU* | 58(132) | 36 | 0.00E+00 | 57.62 |
| *FBGS/+; UAS fatp^RNAi^/+ DR +RU* | 86(100) | 46 |  |  |
| *FBGS/+; UAS fatp^RNAi^/+ AL -RU* | 58(85) | 36 | 0.0746 | 3.18 |
| *FBGS/+; UAS fatp^RNAi^/+ AL +RU* | 58(132) | 36 |  |  |
| *FBGS/+; UAS fatp^RNAi^/+ DR -RU* | 96(79) | 54 | 0.0002 | 13.49 |
| *FBGS/+; UAS fatp^RNAi^/+ DR +RU* | 86(100) | 46 |  |  |

^#^Experiment 2 is represented in Figure 7J; p value calculated by log rank test; χ^2^, Chi^2^ calculated by Log rank test.

**Figure 7-source data 2B.** Cox regression analysis of *FBGS> UAS fatp1^RNAi^*.

|  | **Risk factor** | **p value** |
| --- | --- | --- |
| Experiment 1 | Diet | 0.000058 |
|  | Ligand | 0.021334 |
| Experiment 2^#^ | Diet | 0.000001 |
|  | Ligand | 0.224125 |

**Figure 7-source data 2C.** Lifespan analysis of *+/+; UAS fatp^RNAi^/+* genotype.

|  | **Lifespan (Days)** | | **p value** | **χ^2^** |
| --- | --- | --- | --- | --- |
| ^#^Experiment | Maximum (Number of flies) | Median |  |  |
| *+/+; UAS fatp^RNAi^/+ AL -RU* | 48(83) | 36 | 0.0001 | 16.35 |
| *+/+; UAS fatp^RNAi^/+ DR -RU* | 64(93) | 38 |  |  |
| *+/+; UAS fatp^RNAi^/+ AL +RU* | 44(86) | 32 | 0.00E+00 | 71.68 |
| *+/+; UAS fatp^RNAi^/+ DR +RU* | 62(94) | 42 |  |  |
| *+/+; UAS fatp^RNAi^/+ AL -RU* | 48(83) | 36 | 0.0001 | 14.5 |
| *+/+; UAS fatp^RNAi^/+ AL +RU* | 44(86) | 32 |  |  |
| *+/+; UAS fatp^RNAi^/+ DR -RU* | 64(93) | 38 | 0.927 | 0.01 |
| *+/+; UAS fatp^RNAi^/+ DR +RU* | 62(94) | 42 |  |  |

^#^Experiment is represented in Figure 7-figure supplement 3B.

**Figure 7-source data 2D.** Cox regression analysis of *+/+; UAS fatp^RNAi^/+*

|  | **Risk factor** | **p value** |
| --- | --- | --- |
| Experiment 1 | Diet | 0.00058 |
|  | Ligand | 0.4602 |
