# Supplementary material for: miR-125-chinmo pathway regulates dietary restriction-dependent enhancement of lifespan in Drosophila
Source: eLife. 2021 Jun 8;10:e62621. doi: 10.7554/eLife.62621 (PMC8233039; doi:10.7554/eLife.62621)
Supplement: Figure 7—source data 3. [file elife-62621-fig7-data3.docx]

**Figure 7-source data 3A.** Lifespan analysis of *FBGS/+; UAS Flag fatp/+ flies.*

|  | **Lifespan (Days)** | | **p value** | **χ^2^** |
| --- | --- | --- | --- | --- |
| Experiment 1 | Maximum (Number of flies) | Median |  |  |
| *FBGS/+; UAS Flag fatp/+ AL -RU* | 42(149) | 26 | 0.00E+00 | 40.61 |
| *FBGS/+; UAS Flag fatp/+ DR -RU* | 60(175) | 30 |  |  |
| *FBGS/+; UAS Flag fatp/+ AL +RU* | 50(173) | 32 | 0.00E+00 | 137.39 |
| *FBGS/+; UAS Flag fatp/+ DR +RU* | 70(184) | 44 |  |  |
| *FBGS/+; UAS Flag fatp/+ AL -RU* | 42(149) | 26 | 5.10E-09 | 34.13 |
| *FBGS/+; UAS Flag fatp/+ AL +RU* | 50(173) | 32 |  |  |
| *FBGS/+; UAS Flag fatp/+ DR -RU* | 60(175) | 30 | 0.00E+00 | 79.83 |
| *FBGS/+; UAS Flag fatp/+ DR +RU* | 70(184) | 44 |  |  |
| ^#^Experiment 2 |  |  |  |  |
| *FBGS/+; UAS Flag fatp/+ AL -RU* | 36(120) | 24 | 0.00E+00 | 184.77 |
| *FBGS/+; UAS Flag fatp/+ DR -RU* | 76(114) | 48 |  |  |
| *FBGS/+; UAS Flag fatp/+ AL +RU* | 56(118) | 28 | 0.00E+00 | 190.39 |
| *FBGS/+; UAS Flag fatp/+ DR +RU* | 102(120) | 64 |  |  |
| *FBGS/+; UAS Flag fatp/+ AL -RU* | 36(120) | 24 | 1.50E-08 | 32.07 |
| *FBGS/+; UAS Flag fatp/+ AL +RU* | 56(118) | 28 |  |  |
| *FBGS/+; UAS Flag fatp/+ DR -RU* | 76(114) | 48 | 0.00E+00 | 75.01 |
| *FBGS/+; UAS Flag fatp/+ DR +RU* | 102(120) | 64 |  |  |

^#^Experiment 2 is represented in Figure 7K; p value calculated by log rank test; ** **χ^2^, Chi^2^ calculated by Log rank test.

**Figure 7-source data 3B.** Cox regression analysis of *FBGS/+; UAS Flag fatp/+* flies.

|  | **Risk factor** | **p value** |
| --- | --- | --- |
| Experiment 1 | Diet | 0.00023 |
|  | Ligand | 0.046 |
| Experiment 2^#^ | Diet | 0.0 |
|  | Ligand | 0.000181 |

**Figure 7-source data 3C.** Lifespan analysis of *+/+; UAS Flag fatp/+* flies.

|  | **Lifespan (Days)** | | **p value** | **χ^2^** |
| --- | --- | --- | --- | --- |
| ^#^Experiment 1 | Maximum  (Number of flies) | Median |  |  |
| *+/+; UAS Flag fatp/+* AL -RU | 50(78) | 26 | 0.00E+00 | 85.17 |
| *+/+; UAS Flag fatp/+* DR -RU | 90(98) | 46 |  |  |
| *+/+; UAS Flag fatp/+* AL +RU | 54(105) | 32 | 0.00E+00 | 55.67 |
| *+/+; UAS Flag fatp/+* DR +RU | 90(85) | 48 |  |  |
| *+/+; UAS Flag fatp/+* AL -RU | 50(78) | 26 | 0.0068 | 7.324 |
| *+/+; UAS Flag fatp/+* AL +RU | 54(105) | 32 |  |  |
| *+/+; UAS Flag fatp/+* DR -RU | 90(98) | 46 | 0.9242 | 0.00905 |
| *+/+; UAS Flag fatp/+* DR +RU | 90(85) | 48 |  |  |

**Figure 7-source data 3D.** Cox regression analysis of *+/+; UAS Flag fatp/+* flies.

| **Genotype** | **Risk factor** | **p value** |
| --- | --- | --- |
| *+/+; UAS Flag fatp/+* | Diet | 0 |
|  | Ligand | 0.770831 |
