# Supplementary material for: miR-125-chinmo pathway regulates dietary restriction-dependent enhancement of lifespan in Drosophila
Source: eLife. 2021 Jun 8;10:e62621. doi: 10.7554/eLife.62621 (PMC8233039; doi:10.7554/eLife.62621)
Supplement: Figure 8—source data 1. [file elife-62621-fig8-data1.docx]

**Figure 8-source data 1A.** Lifespan analysis of *FB GS> UAS pri hsa miR-125b-1*

strain.

|  | **Lifespan (Days)** | | **p value** | **χ^2^** |
| --- | --- | --- | --- | --- |
| ^#^Experiment 1 | Maximum (Number of flies) | Median |  |  |
| *FB GS/+; UAS pri hsmiR-125/+ AL-RU* | 46(93) | 32 | 3.90E-05 | 16.91 |
| *FB GS/+; UAS pri hsmiR-125/+ AL+RU* | 66(145) | 34 |  |  |
| *FB GS/+; UAS pri hsmiR-125/+ DR-RU* | 74(99) | 38 | 1.40E-07 | 27.71 |
| *FB GS/+; UAS pri hsmiR-125/+ DR+RU* | 92(135) | 44 |  |  |
| *FB GS/+; UAS pri hsmiR-125/+ AL-RU* | 46(93) | 32 | 2.90E-07 | 26.29 |
| *FB GS/+; UAS pri hsmiR-125/+ DR-RU* | 74(99) | 38 |  |  |
| *FB GS/+; UAS pri hsmiR-125/+ AL+RU* | 88(145) | 34 | 0.00E+00 | 62.17 |
| *FB GS/+; UAS pri hsmiR-125/+ DR+RU* | 92(135) | 44 |  |  |
|  |  |  |  |  |
| Experiment 2 |  |  |  |  |
| *FB GS/+; UAS pri hsmiR-125/+ AL-RU* | 54(195) | 30 | 5.80E-09 | 33.90 |
| *FB GS/+; UAS pri hsmiR-125/+ AL+RU* | 68(229) | 34 |  |  |
| *FB GS/+; UAS pri hsmiR-125/+ DR-RU* | 84(220) | 42 | 2.80E-09 | 35.38 |
| *FB GS/+; UAS pri hsmiR-125/+ DR+RU* | 96(254) | 48 |  |  |
| *FB GS/+; UAS pri hsmiR-125/+ AL-RU* | 54(195) | 30 | 0.00E+00 | 124. 6 |
| *FB GS/+; UAS pri hsmiR-125/+ DR-RU* | 84(220) | 42 |  |  |
| *FB GS/+; UAS pri hsmiR-125/+ AL+RU* | 68(229) | 34 | 0.00E+00 | 101 |
| *FB GS/+; UAS pri hsmiR-125/+ DR+RU* | 96(254) | 48 |  |  |

^#^Experiment 1 is represented in Figure 7; p value calculated by log rank test; χ^2^, Chi^2^ calculated by Log rank test.

**Figure 8-source data 1B.** Cox regression analysis of *FB GS> UAS pri hsa miR-125b-1* strain.

|  | **Risk factor** | **p value** |
| --- | --- | --- |
| Experiment 1 | Diet | 0.000009 |
|  | Ligand | 0.000919 |
| Experiment 2 | Diet | 0.000003 |
|  | Ligand | 0.016425 |
