# Supplementary material for: miR-125-chinmo pathway regulates dietary restriction-dependent enhancement of lifespan in Drosophila
Source: eLife. 2021 Jun 8;10:e62621. doi: 10.7554/eLife.62621 (PMC8233039; doi:10.7554/eLife.62621)
Supplement: Figure 8—figure supplement 1—source data 1. [file elife-62621-fig8-figsupp1-data1.docx]

**Figure 8-figure supplement 1-source data 1A.** Lifespan analysis of flies expressing a single copy of the *UAS hsa miR-125b-1* transgene.

| **Genotype** | **Lifespan (Days)** | | **p value**** | **χ^2^*** |
| --- | --- | --- | --- | --- |
| Experiment 1 | Maximum  (Number of flies) | Median |  |  |
| *+/+, UAS pri hsmiR-125/+ AL -RU* | 36(83) | 26 | 0.001 | 10.86 |
| *+/+, UAS pri hsmiR-125/+ AL +RU* | 38(83) | 26 |  |  |
| *+/+, UAS pri hsmiR-125/+ DR -RU* | 54(128) | 34 | 0.6003 | 0.2746 |
| *+/+, UAS pri hsmiR-125/+ DR +RU* | 58(127) | 34 |  |  |
| *+/+, UAS pri hsmiR-125/+ AL -RU* | 36(83) | 26 | 0.00E+00 | 52.26 |
| *+/+, UAS pri hsmiR-125/+ DR -RU* | 54(128) | 34 |  |  |
| *+/+, UAS pri hsmiR-125/+ AL +RU* | 38(83) | 26 | 0.00E+00 | 37.64 |
| *+/+, UAS pri hsmiR-125/+ DR +RU* | 58(127) | 34 |  |  |
|  |  |  |  |  |
| ^#^Experiment 2 |  |  |  |  |
| *+/+, UAS pri hsmiR-125/+ AL -RU* | 54(126) | 30 | 0.4269 | 0.6311 |
| *+/+, UAS pri hsmiR-125/+ AL +RU* | 56(126) | 28 |  |  |
| *+/+, UAS pri hsmiR-125/+ DR -RU* | 70(123) | 52 | 0.0576 | 3.61 |
| *+/+, UAS pri hsmiR-125/+ DR +RU* | 72(122) | 46 |  |  |
| *+/+, UAS pri hsmiR-125/+ AL -RU* | 54(126) | 30 | 0.0E+00 | 198.15 |
| *+/+, UAS pri hsmiR-125/+ DR -RU* | 70(123) | 52 |  |  |
| *+/+, UAS pri hsmiR-125/+ AL +RU* | 56(126) | 28 | 0.0E+00 | 117.32 |
| *+/+, UAS pri hsmiR-125/+ DR +RU* | 72(122) | 46 |  |  |

^#^Experiment 2 is represented in Figure 8-figure supplement.; p value calculated by log rank test; χ^2^, Chi^2^ calculated by Log rank test.

**Figure 8-figure supplement 1-source data 2B.**  Cox regression analysis of flies expressing a single copy of the *UAS hsa miR-125b-1* transgene.

| **Genotype** | **Risk factor** | **p value** |
| --- | --- | --- |
| Experiment 1 | | |
| *+/+, UAS pri hsmiR-125/+* | Diet | 0.000184 |
|  | Ligand | 0.386453 |
| ^#^Experiment 2 | | |
| *+/+, UAS pri hsmiR-125/+* | Diet | 0.00114 |
|  | Ligand | 0.587906 |

^#^Experiment 2 is represented in Figure 8-figure supplement.
