# Supplementary material for: miR-125-chinmo pathway regulates dietary restriction-dependent enhancement of lifespan in Drosophila
Source: eLife. 2021 Jun 8;10:e62621. doi: 10.7554/eLife.62621 (PMC8233039; doi:10.7554/eLife.62621)
Supplement: Supplementary file 1. [file elife-62621-supp1.docx]

**Supplementary File 1. Genotypes used in this study.**

| **Figure** | **Strain Name** | **Genotype** |
| --- | --- | --- |
| 1B-D, 1-supp 2A, 3A-E, 3-supp 1A-H, 4-supp 2A-I | *w^1118^* | *w^1118^* |
| 1E-G, 1I, 1-supp 2B-D | *let-7-C^hyp^/ Δlet-7C* | *w^1118^; let-7-C^GKI^/ let-7-C^KO2^, P{neoFRT}40A; P{w+, let-7-Cp^3.3kb^::cDNA}VK00033 / {v+, let-7-C ^Δlet-7-C miRNAs^}attP2* |
| 1E-H, 1-supp 2B-D | *let-7-C^hyp^/ Rescue* | *w^1118^; let-7-C^GKI^/ let-7-C^KO2^, P{neoFRT}40A; P{w+, let-7-Cp^3.3kb^::cDNA}VK00033 / {v+, let-7-C}attP2* |
| 1J, 2B, 2G-I | *Rescue (let-7-C^null^ rescue)* | *w^1118^; let-7-C^GKI^ / let-7-C^KO2^, P{neoFRT}40A; {v+, let-7-C} attP2 /+* |
| 1K | *ΔmiR-100* | *w^1118^; let-7-C^GKI^ / let-7-C^KO2^, P{neoFRT}40A; {v+, let-7-C ^ΔmiR-100^} attP2/+* |
| 1L | *Δlet-7* | *w^1118^; let-7-C^GKI^ / let-7-C^KO2^, P{neoFRT}40A; {v+, let-7-C ^Δlet-7^} attP2/+* |
| 1M, 2B, 2G-I | *ΔmiR-125* | *w^1118^; let-7-C^GKI^ / let-7-C^KO2^, P{neoFRT}40A; {v+, let-7-C ^ΔmiR-125^} attP2/+* |
| 1-supp 2E | *let-7-C^KO2^/+* | *w^1118^; let-7-C^KO2^, P{neoFRT}40A /+; +/+* |
| 1-supp 2F | *let-7-C^GKI^/+* | *w^1118^; let-7-C^GKI^/+; +/+* |
| 1-supp 2G | *let-7-C^KO2^/+; let-7-C^hyp^/+* | *w^1118^; let-7-C^GKI^/ let-7-C^KO2^, P{neoFRT}40A; P{w+, let-7-Cp^3.3kb^::cDNA}VK00033 /+* |
| 1-supp 2H | *let-7-C^GKI^/+; let-7-C/+* | *w^1118^; let-7-C^GKI^/+; {v+, let-7-C} attP2 / +* |
| 1-supp 2I | *let-7-C^GKI^/+; ΔmiR-100 /+* | *w^1118^; let-7-C^GKI^/+; {v+, let-7-C ^ΔmiR-100^} attP2 / +* |
| 1-supp 2J | *let-7-C^GKI^/+; Δlet-7 /+* | *w^1118^; let-7-C^GKI^/+; {v+, let-7-C ^Δlet-7^} attP2 / +* |
| 1-supp 2K | *let-7-C^GKI^/+; ΔmiR-125 /+* | *w^1118^; let-7-C^GKI^/+; {v+, let-7-C ^ΔmiR-125^} attP2 / +* |
| 1-supp 2L | *let-7-C^GKI^/+; Δlet-7-C/+* | *w^1118^; let-7-C^GKI^/+; {v+, let-7-C ^Δlet-7-C^} attP2 / +* |
| 2C, 2G-I | *Rescue/chinmo^RNAi^* | *w^1118^; let-7-C^GKI^ / let-7-C^KO2^, P{neoFRT}40A; {v+, let-7-C} attP2 / P{w+, UAS-chinmo^RNAi 148^}VK00033* |
| 2D, 2G-I | *ΔmiR-125, chinmo^RNAi^* | *w^1118^; let-7-C^GKI^ / let-7-C^KO2^, P{neoFRT}40A; {v+, let-7-C ^ΔmiR-125^} attP2 / P{w+, UAS-chinmo^RNAi 148^}VK00033* |
| 2E, 2G-I | *chin^1^, rescue* | *w^1118^; let-7-C^GKI^ / chinmo^1^, let-7-C^KO2^, P{neoFRT}40A; {v+, let-7-C} attP2 /+* |
| 2F, 2G-I | *chin^1^, ΔmiR-125* | *w^1118^; let-7-C^GKI^ / chinmo^1^, let-7-C^KO2^, P{neoFRT}40A; {v+, let-7-C ^ΔmiR-125^ } attP2 / +* |
| 2-supp 1A | *UAS-chinmo^RNAi^/+* | *w^1118^;+/+; P{w+, UAS-chinmo^RNAi 148^}VK00033/+* |
| 2-supp 1B | *chin^1^, let-7-C^KO2^/+* | *w^1118^; chinmo^1^, let-7-C^KO2^, P{neoFRT}40A/+; +/+* |
| 3F-J | *FB-GS, UAS chinmo^RNAi^* | *w^1118^; P{w[+mW.hs]=Switch1}106/+; P{w+, UAS-chinmo^RNAi 148^}VK00033/+* |
| 6A-B, 6F, 6H,6J-K, 6-supp 1H, 6-supp 2I-P, 7A-H, 7-supp 1A-E | *3XelavGS*>*UAS-chinmo* | *P{elav-Switch.O}GS -1A / +; P{elav-Switch.O}GS-3A/+, P{elav-Switch.O} GSG301 / P{w+, UAS-chin::SV40}/+* |
| 6C,6H,6L-M, 6-supp 1D-G, 6-supp 2I’-P’ | *FB-GS, UAS-chinmo* | *w^1118^; P{w[+mW.hs]=Switch1}106/+; P{w+, UAS-chin::SV40}/+* |
| 6D, 6G, 6I, 6-supp 1I, 6-supp 3A-E, 7-supp 3A | *3XElavGS*>*UAS Flag chinmo* | *P{elav-Switch.O}GS -1A / +; P{elav-Switch.O}GS-3A/+, P{elav-Switch.O} GSG301 / P{w+, UAS-Flag chin::SV40} attP2 / +* |
| 6E | *FB-GS, UAS Flag chinmo* | *w^1118^; P{w[+mW.hs]=Switch1}106/+; P{w+, UAS-chin::SV40} attP2 / +* |
| 6-supp 1A | *3XElav GS/+; ElavGS, ElavGS/+* | *P{elav-Switch.O}GS -1A / +; P{elav-Switch.O}GS-3A/+, P{elav-Switch.O} GSG301 /+* |
| 6-supp 1B | *UAS-chinmo/+* | *w^1118^; +/+; P{w+, UAS-chin::SV40}/+* |
| 6-supp 1C | *UAS Flag chinmo/+* | *w^1118^; +/+; P{w+, UAS-Flag chin::SV40} attP2 /+* |
| 6-supp 2A-H | *3XElavGS*>*UAS GFP* | *P{elav-Switch.O}GS -1A / +; P{elav-Switch.O}GS-3A, P{elav-Switch.O} GSG301/P{y[+t7.7] w[+mC]=10XUAS-IVS-myr::GFP}attP40* |
| 6-supp 2A’-H’ | *FBGS*>*UAS GFP* | *w^1118^; P{w[+mW.hs]=Switch1}106/ P{y[+t7.7] w[+mC]=10XUAS-IVS-myr::GFP}attP40; +/+* |
| 7I, 7-supp 3B | *FB GS, UAS fasn1^RNAi^/+* | *w^1118^; P{w[+mW.hs]=Switch1}106/+; P{y[+t7.7] v[+t1.8]=TRiP.HMS01524}attP2/+* |
| 7J, 7-supp 3C | *FB GS/+; UAS fatp^RNAi^/+* | *w[1118]; P{w[+mW.hs]=Switch1}106/+; P{y[+t7.7] v[+t1.8]=TRiP.HMC04206}attP2/+* |
| 7K, 7-supp 3F | *FB GS/+; UAS Flag fatp/+* | *w[1118]; P{w[+mW.hs]=Switch1}106/+;P{w+, UAS-Flag FATP} attP2 / +* |
| 7-supp 3A | *UAS fasn1^RNAi^/+* | *w^1118^;+/+; P{y[+t7.7] v[+t1.8]=TRiP.HMS01524}attP2/+* |
| 7-supp 3B | *UAS fatp^RNAi^/+* | *w^1118^;+/+; P{y[+t7.7] v[+t1.8]=TRiP.HMC04206}attP2/+* |
| 7-supp 3C | *UAS Flag fatp/+* | *w^1118^;+/+; P{w+, UAS-Flag FATP} attP2 / +* |
| 8B-H | *FB GS, UAS hsa miR-125b-1* | *w^1118^; P{w[+mW.hs]=Switch1}106/+; P{v+, UAS-hsa miR-125b-1} VK00033/+* |
| 8-supp 1 | *UAS has miR-125b-1/+* | *w^1118^; +/+; P{v+, UAS-hsa miR-125b-1} VK00033/+* |
