# Supplementary material for: miR-125-chinmo pathway regulates dietary restriction-dependent enhancement of lifespan in Drosophila
Source: eLife. 2021 Jun 8;10:e62621. doi: 10.7554/eLife.62621 (PMC8233039; doi:10.7554/eLife.62621)
Supplement: Supplementary file 2. [file elife-62621-supp2.docx]

**Supplementary File 2. Primers used in this study.**

| No. | Name | Sequence |
| --- | --- | --- |
| 7 | Chinmo QPCR For | AGTTCTGCCTCAAATGGAACAG |
| 8 | Chinmo QPCR Rev | CGCAGGATAATATGACATCGGC |
| 23 | act-5c QPCR For | CACACCAAATCTTACAAAATGTGT |
| 24 | act-5c QPCR Rev | AATCCGGCCTTGCACATG |
| 102a | FASN 1 RT PCR For | GTGCGTCCTATCAGCTACCC |
| 103a | FASN 1 RT PCR Rev | GTCTGCCAAGCCAGAGTCAT |
| 1004 | CHUTRSHRNA.3T(NS) | ctagcagtccaactgaatttcaattgtgatagttatattcaagcatatcacaattgaaattcagttgggcg |
| 1005 | CHUTRSHRNA.3B(NS) | aattcgcccaactgaatttcaattgtgatatgcttgaatataactatcacaattgaaattcagttggactg |
| 179 | FATP RT-PCR For | GCGGTTATCTCTCACTCCC |
| 180 | FATP RT-PCR Rev | CGCAGTCGGCGAAATAGTTG |
| 181 | CG2107 RT PCR For | CCACACGGGACTTCTGTGAA |
| 182 | CG2107 RT PCR Rev | ATGCGCTTGTAAGCCTCAGT |
| 183 | CG5009 RT PCR For | AGACCAGGGCTGACTACGAT |
| 184 | CG5009 RT PCR Rev | CATGGGACGGTGAGTATCGG |
| 185 | CG8778 RT PCR For | AGAGCGTCTGGTTTAGCTCG |
| 186 | CG8778 RT PCR Rev | CCTCGGGAGTCATGCCTTTT |
| 187 | CG9527 RT PCR For | ACTTCCGTAGCGGACCTTTG |
| 188 | CG9527 RT PCR Rev | GCAGAAGATGTGGGGTTCCA |
| 189 | CG9577 RT PCR For | GACTGGCCACTAATCCCGAC |
| 190 | CG9577 RT PCR Rev | CCGATGTCCACCTCCTTGAC |
| 191 | CG17544 RT PCR For | GTGCCCAAGGAGATCGAGAG |
| 192 | CG17544 RT PCR Rev | GTGTTGCTGCCATGCGATAG |
| 193 | CG10467 RT-PCR For | GCCGTATCACACCCGTAGAG |
| 194 | CG10467 RT-PCR Rev | GTTCACGGAGTCGGGAAACT |
| 003 | Rp-49 QPCR For | CCCAAGGGTATCGACAACAGA |
| 004 | Rp-49 QPCR Rev | CGATGTTGGGCATCAGATACTG |
| 115 | EcoR1 3X Flag FASN1 For | CGGAATTCCCATGGACTACAAAGACCATGACGGTGATTATAAAGATCATGACATCGATTACAAGGATGACGATGACAAGCCCGCCCGATTCGCCGAGGA |
| 114 | Xba1 FASN1 cDNA Rev | gatctctagaTTAGTTGAACAGACGCTTCAG |
| 211 | Xba 1 Fatp cDNA 3X Flag Rev | GATCtctagaTTACTTGTCGTCATCGTCTTTGTAGTCGATGTCATGATCTTTATAATCACCGTCATGGTCTTTGTAGTCgaagcggatttcgttgcgctg |
| 209 | Xho1 Fatp 1.8kb cDNA pUASTattB For | GATCctcgagCACCATGGGCTGGATTTTTGCTGTGCTCG |
| 1072(NS) | hsa miR-125b1 For | ctagcAACATTGTTGCGCTCCTCTCAGTCCCTGAGACCCTAACTTGTGATGTTTACCGTTTAAATCCACGGGTTAGGCTCTTGGGAGCTGCGAGTCGTGCTTTTGCATCCTGGAAg |
| 1073  (NS) | hsa miR-125b1 Rev | CTTCCAGGATGCAAAAGCACGACTCGCAGCTCCCAAGAGCCTAACCCGTGGATTTAAACGGTAAACATCACAAGTTAGGGTCTCAGGGACTGAGAGGAGCGCAACAATGTTGCTAG |
| 109 | EcoR1 3X Flag Chinmo For | CGGAATTCCCATGGACTACAAAGACCATGACGGTGATTATAAAGATCATGACATCGATTACAAGGATGACGATGACAAGGATCCGCAGCAGCAGTTCTGCC |
| 108 | Xho 1 Chinmo cDNA Rev | GATCctcgagCTATGGTGAATGATTGCTGGCTG |
| 241 | Chinmo cDNA Xho I For | GATCctcgagGATCCGCAGCAGCAGTTCTG |
| 242 | Chinmo cDNA Xba I Rev | gatctctagaCTATGGTGAATGATTGCTGGCTG |
| 237 | EcoRI Opt GFP For | gatcGAATTCACCGTGTCCAAGGGCGAGGAGCTGTTC |
| 238 | Xho I Opt GFP Rev | GATCctcgagGTACAGCTCATCCATGCCCAG |
| 328 | Lys-Gln 358 Chinmo Rev | CGGTGGTACGGCGGCTGGTTGATGGGGCTG |
| 329 | Lys-Gln 358 Chinmo For | CAGCCCCATCAACCAGCCGCCGTACCACCG |
| 330 | Lys-Gln 414 Chinmo Rev | CCATTGTTTCGCTCCGATTGCACAATGCGCTCC |
| 331 | Lys-Gln 414 Chinmo For | GGAGCGCATTGTGCAATCGGAGCGAAACAATGG |
| 332 | Lys-Gln 506 Chinmo Rev | GTTCACGCTGTTCTGGTTGTTCATCTTGGTG |
| 333 | Lys-Gln 506 Chinmo For | CACCAAGATGAACAACCAGAACAGCGTGAAC |
| 356 | Lys-Gln 27 Chinmo Rev | GCCAGTAGATCCGATTGGAACAGATTGGAG |
| 357 | Lys-Gln 27 Chinmo For | CTCCAATCTGTTCCAATCGGATCTACTGGC |
| 358 | Lys-Gln 47 Chinmo Rev | CGCCAATATAAGTTGGTGGGCTTTGAATAC |
| 359 | Lys-Gln 47 Chinmo For | GTATTCAAAGCCCACCAACTTATATTGGCG |
| 360 | Lys-Gln 55 Chinmo Rev | AGATCGGCGAACTTCTGTGAGCAGGCCGCC |
| 361 | Lys-Gln 55 Chinmo For | GGCGGCCTGCTCACAGAAGTTCGCCGATCT |
| 362 | Lys-Gln 114 Chinmo Rev | CCGTGGACAGACCTTGGACCTGCAAACTCT |
| 363 | Lys-Gln 114 Chinmo For | AGAGTTTGCAGGTCCAAGGTCTGTCCACGG |
| 364 | Lys-Gln 288 Chinmo Rev | AGTCGCATTCCTGCTGCAAGATGCCAGTGG |
| 365 | Lys-Gln 288 Chinmo For | CCACTGGCATCTTGCAGCAGGAATGCGACT |
| 366 | Lys-Gln 348 Chinmo Rev | GGACACTCGGAGGACTGGGAGCCATCTCGC |
| 367 | Lys-Gln 348 Chinmo For | GCGAGATGGCTCCCAGTCCTCCGAGTGTCC |
| 368 | Lys-Gln 515 Chinmo Rev | AGACACTTCAGCTTCTGACCGTCGGCCGTG |
| 369 | Lys-Gln 515 Chinmo For | CACGGCCGACGGTCAGAAGCTGAAGTGTCT |
| 370 | Lys-Gln 569 Chinmo Rev | CAATCCGATTTCCTGCTGGTGCTCCCTGGC |
| 371 | Lys-Gln 569 Chinmo For | GCCAGGGAGCACCAGCAGGAAATCGGATTG |
| 322 | Flag dSir2 Not 1 For | GATCGCGGCCGCCCATGGACTACAAAGACCATGACGGTGATTATAAAGATCATGACATCGATTACAAGGATGACGATGACAAGATGGAAAATTACGAGGAAATTC |
| 323 | Xba1 dSir2 cDNA Rev | ATCtctagaTTACACTGCTGCTAACTGTCC |
| 586 (JT) | pENTR dTOPO Flag-N dSir2 For | CACCatggaaaattacgaggaaattcg |
| 587  (JT) | pENTR dTOPO Flag-N dSir2 Rev | ttacgtttacactgctgctaactgtcctg |
