# Supplementary material for: miR-125-chinmo pathway regulates dietary restriction-dependent enhancement of lifespan in Drosophila
Source: eLife. 2021 Jun 8;10:e62621. doi: 10.7554/eLife.62621 (PMC8233039; doi:10.7554/eLife.62621)
Supplement: Transparent reporting form [file elife-62621-transrepform.docx]

***eLife’s* transparent reporting form**

We encourage authors to provide detailed information *within their submission* to facilitate the interpretation and replication of experiments. Authors can upload supporting documentation to indicate the use of appropriate reporting guidelines for health-related research (see [EQUATOR Network](http://www.equator-network.org/%20)), life science research (see the [BioSharing Information Resource](https://biosharing.org/)), or the [ARRIVE guidelines](http://www.plosbiology.org/article/info:doi/10.1371/journal.pbio.1000412) for reporting work involving animal research. Where applicable, authors should refer to any relevant reporting standards documents in this form.

If you have any questions, please consult our Journal Policies and/or contact us: [editorial@elifesciences.org](mailto:editorial@elifesciences.org).

**Sample-size estimation**

- You should state whether an appropriate sample size was computed when the study was being designed
- You should state the statistical method of sample size computation and any required assumptions
- If no explicit power analysis was used, you should describe how you decided what sample (replicate) size (number) to use

Please outline where this information can be found within the submission (e.g., sections or figure legends), or explain why this information doesn’t apply to your submission:

-For lifespan analysis (N=72 -200) flies were used for each group where 50-100 flies allow reasonable inferences to be drawn (Linford et al., 2013). At least two independent replicates were performed for each experiment.

-For control crosses, to test effects of the genetic background lifespan analysis was performed one time after crossing with wild type flies.

-For Real time PCR assays and TAG colorimetric assays, 5-6 biological replicates were used for each group and unpaired t-test with Welch’s correction was performed for the analysis.

-For measuring pixel intensity (immunostaining) at least 10 cells were quantitated per replicate. 5 biological replicates were analysed per group per experiment. Each experiment was performed at least three times.

**Replicates**

- You should report how often each experiment was performed
- You should include a definition of biological versus technical replication
- The data obtained should be provided and sufficient information should be provided to indicate the number of independent biological and/or technical replicates
- If you encountered any outliers, you should describe how these were handled
- Criteria for exclusion/inclusion of data should be clearly stated
- High-throughput sequence data should be uploaded before submission, with a private link for reviewers provided (these are available from both GEO and ArrayExpress)

Please outline where this information can be found within the submission (e.g., sections or figure legends), or explain why this information doesn’t apply to your submission:

-Survivorship curves were used for survival analysis. Sample size for each lifespan assay has been indicated in the source data tables attached to each figure that has lifespan assays. Between all experiments, the number of flies varied between 72-200 per replicate. Equality between the two groups was inferred using log-rank test and p values as well Chi-square values were represented to allow comparison between different experiments.

- Biological replicates in all experiments were biological independent replicates at the same time and same condition that were processed independently (e.g. for RNA extraction, for TAG assay, for immunostaining).

-Individual data points have been plotted in the figure panels for all the real time PCR, TAG, Nile red staining and immunostaining and immunoprecipitation experiments.

-For proteomics analysis-three biological replicates were used for the experiment.

**Statistical reporting**

- Statistical analysis methods should be described and justified
- Raw data should be presented in figures whenever informative to do so (typically when N per group is less than 10)
- For each experiment, you should identify the statistical tests used, exact values of N, definitions of center, methods of multiple test correction, and dispersion and precision measures (e.g., mean, median, SD, SEM, confidence intervals; and, for the major substantive results, a measure of effect size (e.g., Pearson's r, Cohen's d)
- Report exact p-values wherever possible alongside the summary statistics and 95% confidence intervals. These should be reported for all key questions and not only when the p-value is less than 0.05.

Please outline where this information can be found within the submission (e.g., sections or figure legends), or explain why this information doesn’t apply to your submission:

- Statistical analysis methods have been described in the legends and Material methods section.

- All data points are indicated in the figures. Exact p-values have been reported on each figure panel.

- Statistical tests used are indicated in the figure legends and methods. T-test with Welch’s correction was performed for QRT-PCR, TAG assays, LPD diameter and measurement of immunostaining intensity. Mean ± SD have been represented for all experiments. For analysis of lifespans, pairwise comparisons were made and Log rank test was performed to determine p-value.

-P-values are reported for figure panels where statistical analysis was performed.

(For large datasets, or papers with a very large number of statistical tests, you may upload a single table file with tests, Ns, etc., with reference to sections in the manuscript.)

**Group allocation**

- Indicate how samples were allocated into experimental groups (in the case of clinical studies, please specify allocation to treatment method); if randomization was used, please also state if restricted randomization was applied
- Indicate if masking was used during group allocation, data collection and/or data analysis

Please outline where this information can be found within the submission (e.g., sections or figure legends), or explain why this information doesn’t apply to your submission:

-Comparisons were made between Rescue (Control) or mutant (Experiment) lines.

-For studying the effects of over expression or knockdown of specific genes, comparisons were made after crossing GAL4 lines with the UAS overexpression or RNAi lines. Comparisons were made in uninduced (Control) and induced (Experimental) conditions.

-To study the effect of diet, flies of the same genotype were exposed to AL or DR diet.

**Additional data files (“source data”)**

- We encourage you to upload relevant additional data files, such as numerical data that are represented as a graph in a figure, or as a summary table
- Where provided, these should be in the most useful format, and they can be uploaded as “Source data” files linked to a main figure or table
- Include model definition files including the full list of parameters used
- Include code used for data analysis (e.g., R, MatLab)
- Avoid stating that data files are “available upon request”

Please indicate the figures or tables for which source data files have been provided:

-Source data with survival proportions for all lifespan curves is provided as an excel sheet for each figure panel in which survival analysis was done. In additional summary tables indicating the number of flies, p value and chi2 value for pairwise comparison for all replicates are included as source data in a word document table (Fig 1, Fig 2, Fig 3, Fig6, Fig7 and Fig8).

-Raw data is provided in source data file (Fig 4, Fig5 and Fig 7) for all western blots in figures where western blot analysis is represented.
